# SHIP2 inhibition alters redox‐induced PI3K/AKT and MAP kinase pathways via PTEN over‐activation in cervical cancer cells

**DOI:** 10.1002/2211-5463.12967

**Published:** 2020-10-01

**Authors:** Abdelhalim Azzi

**Affiliations:** ^1^ GIGA‐Molecular Biology of Disease GIGA‐B34 University of Liège Belgium

**Keywords:** PI3K/Akt pathway, PTEN, ROS, SHIP2, SHIP2 inhibitor

## Abstract

Phosphatidylinositol (3,4,5)‐trisphosphate (PI(3,4,5)P3) is required for protein kinase B (AKT) activation. The level of PI(3,4,5)P3 is constantly regulated through balanced synthesis by phosphoinositide 3‐kinase (PI3K) and degradation by phosphoinositide phosphatases phosphatase and tensin homologue (PTEN) and SH2‐domain containing phosphatidylinositol‐3,4,5‐trisphosphate 5‐phosphatase 2 (SHIP2), known as negative regulators of AKT. Here, I show that SHIP2 inhibition in cervical cancer cell lines alters H_2_O_2_‐mediated AKT and mitogen‐activated protein kinase/extracellular signal‐regulated kinase pathway activation. In addition, SHIP2 inhibition enhances reactive oxygen species generation. Interestingly, I found that SHIP2 inhibition and H_2_O_2_ treatment enhance lipid and protein phosphatase activity of PTEN. Pharmacological targeting or RNA interference(RNAi) mediated knockdown of PTEN rescues extracellular signal‐regulated kinase and AKT activation. Using a series of pharmacological and biochemical approaches, I provide evidence that crosstalk between SHIP2 and PTEN occurs upon an increase in oxidative stress to modulate the activity of mitogen‐activated protein kinase and phosphoinositide 3/ATK pathways.

## Graphical abstract

Azzi investigated the effects of SH2‐domain containing phosphatidylinositol‐3,4,5‐trisphosphate 5‐phosphatase (SHIP2) inhibition by AS1938909 on H_2_O_2_‐induced phosphoinositide 3‐kinase/protein kinase B and mitogen‐activated protein/extracellular signal‐regulated kinase pathways in two cervical cancer cell lines, HeLa and SiHa. After H_2_O_2_ treatment, SHIP2 inhibition enhanced phosphatase and tensin homologue activity, which, in turn, affected phosphoinositide 3‐kinase/protein kinase B and mitogen‐activated protein kinase/extracellular signal‐regulated kinase activation.

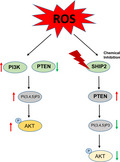

**Abbreviations**
ΔΨm: mitochondrial membrane potential
AKT: protein kinase B
AS19: AS1938909
CCK‐8: Cell Counting Kit‐8
DAPI: 4′,6‐diamidino‐2‐phenylindole dihydrochloride
DCF: 2',7'‐dichlorofluorescein
DCFDA: dichlorofluorescein diacetate
DMEM: Dulbecco's modified Eagle's medium
ER: endoplasmic reticulum
ERK: extracellular signal‐regulated kinase
H_2_O_2_: hydrogen peroxide
MAPK: mitogen‐activated protein kinase
PARP: Poly (ADP‐ribose) polymerase
p‐: phosphorylated
PH: pleckstrin homology
PI(3,4,5)P3: Phosphatidylinositol (3,4,5)‐trisphosphate
PPI(3,4)P2: Phosphatidylinositol (3,4)‐bisphosphate
PI3K: phosphoinositide 3‐kinase
PLCδ: phospholipase C, delta
PTEN: phosphatase and tensin homologue
RNAi: RNA interference
ROS: reactive oxygen species
SEM: standard error of the mean
SHC: Src homology 2 domain‐containing protein
SHIP2: SH2‐domain containing phosphatidylinositol‐3,4,5‐trisphosphate 5‐phosphatase 2

Reactive oxygen species (ROS) play a central role in cellular physiology [1, 2]. At lower concentration, they play a role as a second messenger by regulating diverse aspects of cellular signaling during organismal development [3, 4]. However, at high concentration, ROS alter cellular function by damaging lipids, proteins and DNA, leading to the activation of stress signaling pathways. Activation of these pathways leads to growth arrest to repair damages and promote survival, or activates cell death [5, 6, 7]. Hydrogen peroxide (H_2_O_2_) is the main substance used to study oxidative stress at the cellular level. Experimental evidence exists for H_2_O_2_‐mediated ROS production that activates several signaling pathways, among which are mitogen‐activated protein kinase (MAPK) and phosphatidylinositol 3‐kinase (PI3K)/protein kinase B (Akt), which promote cell growth and survival [8, 9]. Activation of the MAPK family members, extracellular signal‐regulated kinases 1 and 2 (ERK1/2), blocks apoptosis in response to H_2_O_2_ [10]. The antiapoptotic effect of ERK1/2, for example, involves protection against caspase activation and loss of mitochondrial membrane potential (ΔΨm) [11, 12]. In addition to MAPK, AKT activation also blocks apoptosis and enhances survival in response to exogenous H_2_O_2_. The antiapoptotic effect of AKT involves direct phosphorylation and inactivation of proapoptotic proteins [13, 14, 15]. Activation of AKT requires Phosphatidylinositol (3,4,5)‐trisphosphate (PI(3,4,5)P3) [16]. Intracellular levels of PI(3,4,5)P3 are constantly regulated by a balance of two processes that involve synthesis by PI3K and degradation by phosphoinositide phosphatases PTEN (phosphatase and tensin homologue) and SH2‐domain containing phosphatidylinositol‐3,4,5‐trisphosphate 5‐phosphatase (SHIP2) [15, 17]. PTEN dephosphorylates the three‐position phosphate of PI(3,4,5)P3 and Phosphatidylinositol (3,4)‐bisphosphate (PI(3,4)P2) to generate, respectively, PI(4,5)P2 and PI4P, and this event results in suppression of AKT signaling [18]. SHIP2 dephosphorylates the five‐position phosphate from PI(3,4,5)P3 to generate PI(3,4)P2, leading also to inhibition of AKT‐mediated signaling pathways in response to growth factor like insulin [19, 20]. Previous studies have shown that SHIP2 plays a role in regulation of PI3K‐dependent insulin signaling [21, 22, 23]. In addition to its role as a lipid phosphatase, SHIP2 also plays a role as a scaffolding protein in other aspects of signaling, including cell migration, adhesion and endocytosis [24, 25, 26]. Interestingly, recent findings reported that dysregulation in the levels of SHIP2 protein alters cellular health and signaling. For example, overexpression of SHIP2 suppresses AKT activation and promotes apoptosis, whereas its knockdown enhances AKT activation and promotes cell growth [27, 28]. Moreover, SHIP2 is also involved in oxidative stress‐activated signaling. Overexpression of the dominant‐negative form of SHIP2 in HepG2 hepatocellular carcinoma cells suppresses palmitate‐induced apoptosis, enhances AKT activation and reduces ROS generation [29]. However, this study was performed in cells that express lower levels of PTEN. So far, signaling crosstalk between SHIP2 and PTEN in response to oxidative stress has not been a subject of investigation. Here, I used a pharmacological approach to examine whether blocking SHIP2 would promote AKT activation and survival upon H_2_O_2_‐induced oxidative stress in cervical cancer cell lines. Experiments were performed in HeLa and SiHa cells, which express higher levels of both SHIP2 and PTEN [30, 31]. SHIP2 was blocked using 3‐[(4‐chlorobenzyl)oxy]‐*N*‐[(1S)‐1‐phenylethyl]‐2‐thiophene‐carboxamide (AS1949490), a small‐molecule drug that was previously characterized from a high‐throughput screen [32]. The specificity of the inhibitory activity of AS1949490 was validated *in vitro* and *in vivo* [32, 33]. In this study, I report that in response to H_2_O_2_, SHIP2 inhibition enhances lipid and protein phosphatase activity of PTEN, which, in turn, down‐regulates MAPK and PI3K/Akt pathways.

## Results

### Catalytic inhibition of SHIP2 alters PI3K and MAPK activation in stimuli and in a cell‐type‐dependent manner

**Fig. 1:**
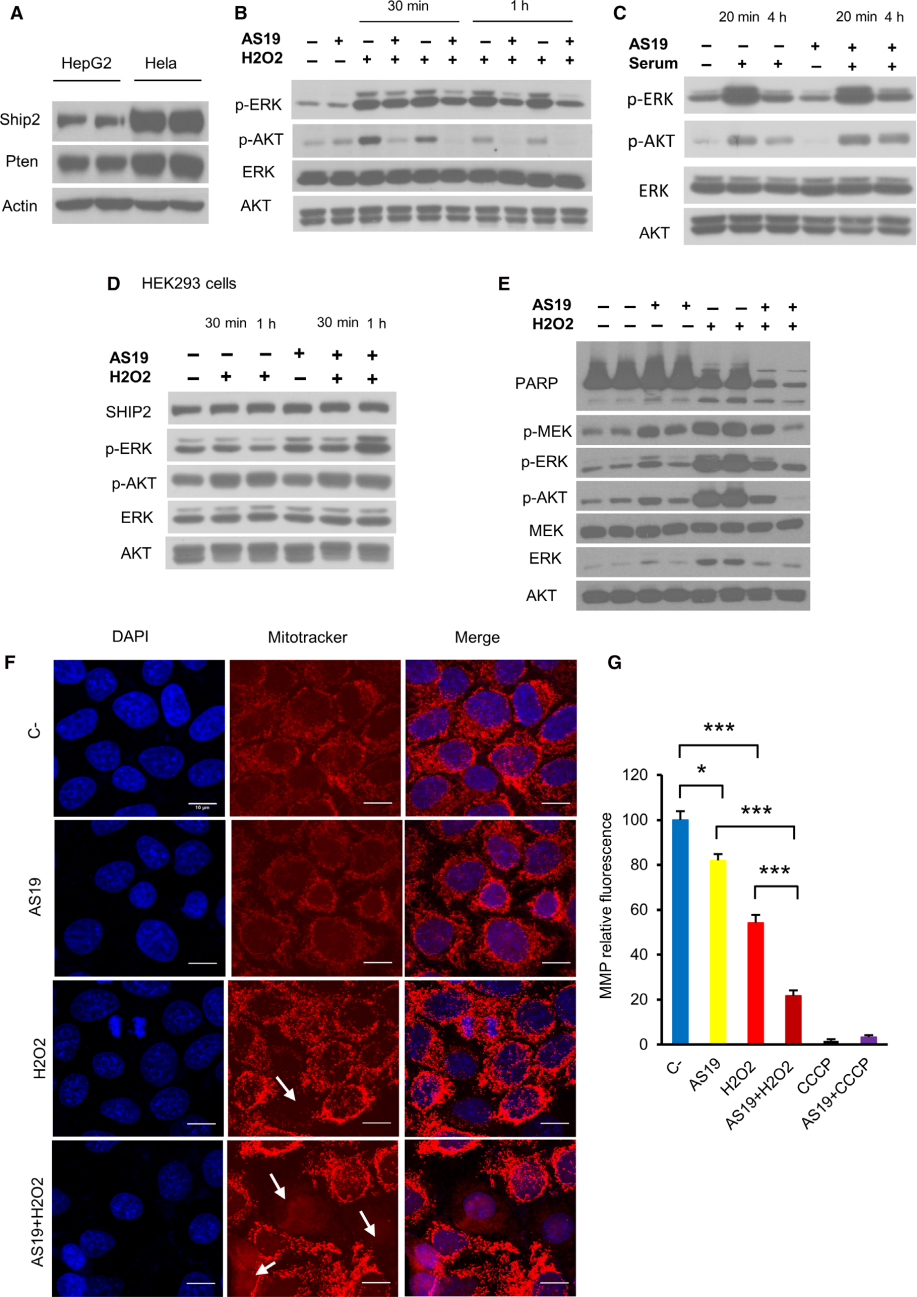
SHIP2 inhibition alters the PI3K and MAPK pathways upon H_2_O_2_ treatment. (A) Lysates from HepG2 and HeLa cells blotted with the indicated antibodies. Results are representative of two experimental replicates. (B) HeLa cells treated with vehicle or SHIP2 inhibitor AS19 for 24 h, then followed by 1 mm H_2_O_2_ for 30 min or 1 h. H_2_O_2_ treatment was performed in two biological replicates for each condition. Lysates were separated using SDS/PAGE and blotted with the indicated antibodies. Results are representative of two independent experiments. (C) HeLa cells treated or not with SHIP2 inhibitor for 24 h followed by 6‐h serum starvation (SS), then 10% serum stimulation for the indicated times. (D) H_2_O_2_‐induced AKT and ERK1/2 phosphorylation upon SHIP2 inhibition is cell type dependent. HEK293T cells treated with vehicle or AS19 for 24 h, then followed by 1 mm H_2_O_2_ for the indicated times. (E) HeLa cells were treated as in (B), then exposed to 4 h H_2_O_2_ (1 mm). H_2_O_2_ treatment was performed in two biological replicates for each condition. (F) HeLa cells were treated as described in (B), then exposed to 3.5 h of H_2_O_2_ (1 mm). Live cells were stained with 100 nm MitoTracker Red for mitochondria (red) for 30 min, then fixed and mounted. Nuclei were stained with DAPI (blue). Scale bar: 10 μm. (G) SHIP2 inhibition alters ΔΨm. Bar graph showing relative MitoLite fluorescence intensity in each condition after 1 mm H_2_O_2_ treatment during 90 min. HeLa cells treated with carbonyl cyanide *m*‐chlorophenyl hydrazone alone were used as positive control (25 µm). Data represent the means ± standard error of the mean (SEM) of three independent experiments. One‐way ANOVA, *F*(3, 60) = 111, *P* < 0.0001. Tukey's multiple comparisons test. C‐, vehicle‐treated cells.

Cervical cancer cell lines express high levels of SHIP2 and PTEN [30, 31] (Fig. 1A and Fig. S1). To test whether SHIP2 plays a role in signaling upon an increase in levels of ROS, I treated HeLa cells with a vehicle or SHIP2 inhibitor AS1938909 (abbreviated as AS19) [32]. Twenty‐four hours later, cells were stimulated with 1 mm H_2_O_2_ for 1 h. As a readout of PI3K and MAPK activation, AKT phosphorylation at Ser473 and ERK1/2 phosphorylation known to be activated by H_2_O_2_ were used to examine these pathways [8, 9]. Western blot analysis showed that in vehicle‐treated cells, H_2_O_2_ induces phosphorylation of both AKT and ERK proteins (Fig. 1B). Unexpectedly, in cells in which SHIP2 was inhibited, phosphorylation of both proteins was less pronounced. To further confirm the inhibitory activity of AS19 [32, 33], I first analyzed its effect in response to other stimuli in HeLa cells. As indicated in Fig. 1C, upon growth factor stimulation, AS19‐mediated SHIP2 inhibition induces sustained activation of AKT, demonstrating that the chemical inhibitor indeed blocks the lipid phosphatase activity of SHIP2. Next, I examined the effect of AS19 in another cell line, human embryonic kidney HEK293T cells. As shown in Fig. 1D, SHIP2 inhibition did not suppress AKT phosphorylation upon H_2_O_2_ treatment. Moreover, SHIP2 inhibition enhances both basal and H_2_O_2_‐induced ERK1/2 phosphorylation. Together, these results show that the difference of activation of the two signaling pathways upon SHIP2 inhibition is both stimuli and cell type dependent. Then I focused on the molecular mechanism that drives this difference in signaling in HeLa cells.

I first examined whether lower AKT and ERK1/2 phosphorylation seen upon SHIP2 inhibition and H_2_O_2_ stimulation is time dependent. Compared with vehicle‐treated cells, longer treatment with H_2_O_2_ did not increase AKT and ERK phosphorylation upon SHIP2 inhibition (Fig. 1E). In addition to these differences in H_2_O_2_ signalling response, Poly (ADP‐ribose) polymerase (PARP) cleavage, known as a marker of apoptosis, was also analyzed [34]. Contrary to previous observation [29], SHIP2 inhibition did not prevent oxidative stress‐induced apoptosis (Fig. 1E). To further confirm that SHIP2 inhibition induces apoptosis in these cells, I verified ΔΨm. ROS‐mediated apoptosis alters mitochondrial dynamics, as well as ΔΨm [35]. I therefore examined whether SHIP2 inhibition alters mitochondrial properties. MitoTracker© Red CMXRos, a fluorescent dye that accumulates specifically in intact mitochondria, was used [36]. Under basal conditions, SHIP2 inhibition has no effect on ΔΨm. However, upon H_2_O_2_ treatment, cells in which SHIP2 is inactivated exhibited a marked decline in the ΔΨm, indicative of apoptosis activation (Fig. 1F,G).

### SHIP2 inhibition alters AKT and ERK phosphorylation through increase in ROS generation

**Fig. 2:**
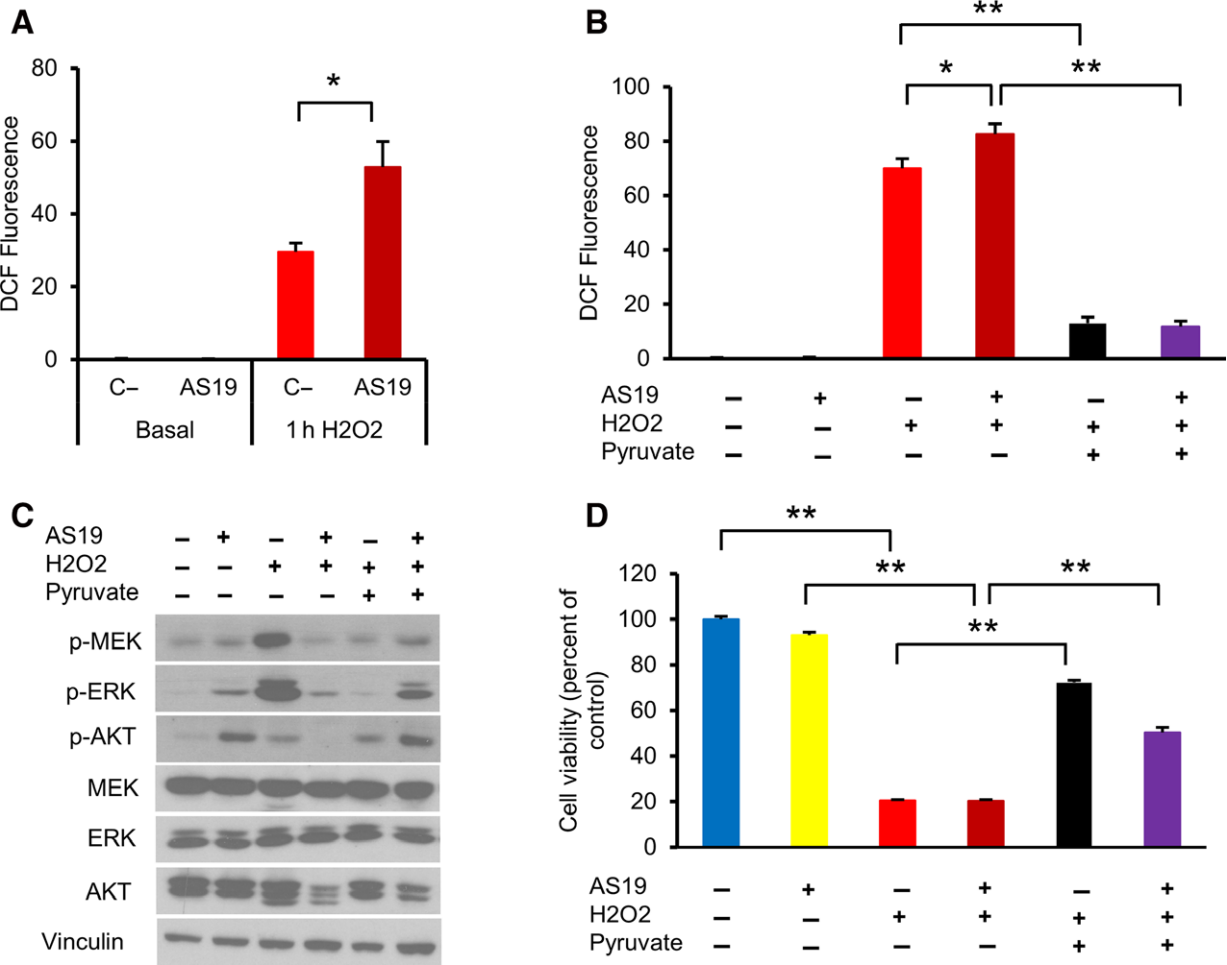
SHIP2 inhibition enhances ROS production upon H_2_O_2_ treatment. (A) Levels of cytoplasmic ROS in HeLa cells were measured by monitoring 2',7'‐dichlorofluorescein (DCF) fluorescence intensity, H_2_O_2_ (1 mm). Bar graph summarizing the DCF intensities for each group, expressed as a fold change relative to control untreated cells. Data are the means ± SEM from three independent experiments. One‐way ANOVA, *F*(3, 28) = 48.13. Tukey's multiple comparisons test (*P ˂ 0.05). C‐, vehicle‐treated cells. (B) Levels of cytoplasmic ROS were measured by monitoring DCF fluorescence intensity. HeLa cells were first treated with vehicle or SHIP2 inhibitor for 24 h, followed by pyruvate (5 mm) for 2 h, and then treated with 1 mm H_2_O_2_ for 1 h. Bar graph summarizing the DCF intensities for each condition. Data are the means ± SEM from three independent experiments. One‐way ANOVA, *F*(5, 90) = 221, *P* < 0.0001. Tukey's multiple comparisons test, *P ˂ 0.05, **P ˂ 0.01. (C) HeLa cells were treated as described in (A). Lysates were analyzed for the indicated antibodies. (D) The ROS scavenger pyruvate prevents H_2_O_2_‐induced cell death. Cell viability was measured with CCK‐8 assays. HeLa cells were treated as in (A). After 4 h of 1 mm H_2_O_2_, cells were then treated with WST‐8 for 1 h at 37 °C. Data are the means ± SEM from three independent experiments. One‐way ANOVA, *F*(5, 66) = 812.4, *P* < 0.0001. Tukey's multiple comparisons test, **P ˂ 0.01.

Previous studies have shown that SHIP2 inhibition leads to lower ROS production in response to diverse stimuli, including palmitate and high glucose [29, 37]. To examine whether the effect of SHIP2 inhibition on AKT and ERK activation is mediated through ROS production, I used the fluorescent probe dichlorofluorescein diacetate (DCFDA) to monitor ROS levels in HeLa cells under different conditions. Even though basal ROS were not different, SHIP2 inhibition in these cells generates significantly higher ROS upon H_2_O_2_ treatment (Fig. 2A). Next, to investigate whether SHIP2 inhibition‐mediated lower ERK and AKT phosphorylation observed earlier is due to a high increase in ROS levels, I used pyruvate as an ROS scavenger [38]. As indicated in Fig. 2B, pretreatment of cells with 5 mm pyruvate led to significant reduction in ROS production. In addition, lowering ROS level reactivated both AKT and ERK phosphorylation and prevented H_2_O_2_‐induced cell death (Fig. 2C,D). Taken together, these demonstrated that SHIP2 inhibition alters ROS production upon H_2_O_2_ treatment. Although less pronounced, similar effects were observed in another cervical cancer cell line, SiHa. Indeed, SHIP2 inhibition in these cells induces lower activation of the PI3K/AKT pathway. Moreover, consistent with the results seen in HeLa cells, pyruvate pretreatment led to marked increase in AKT phosphorylation (Fig. S2A–C).

### SHIP2 inhibition induces overactivation of PTEN upon increase of ROS

**Fig. 3:**
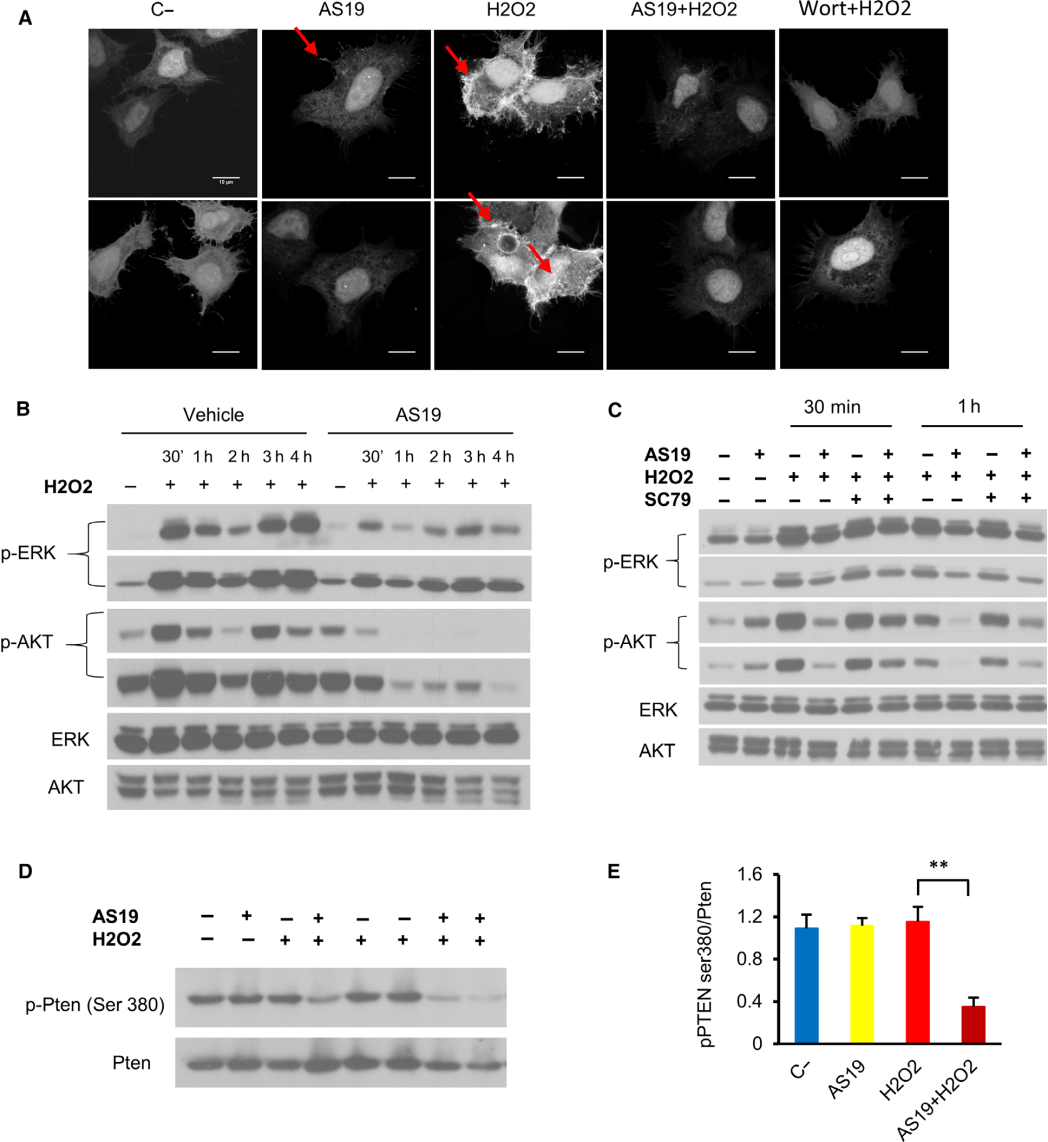
SHIP inhibition alters PI(3,4,5)P3 levels on H_2_O_2_ stimulation. (A) HeLa cells were first transfected with the plasmid GFP‐tagged BTK–PH domain, and 6 h later they were treated with vehicle or AS19 for 24 h. Subsequently, cells were treated with 1 mm H_2_O_2_ for 1 h, then fixed and mounted. Fluorescent images representing PI(3,4,5)P3 levels are shown. Cells pretreated with 200 nm wortmannin (Wort) were used as a control. Arrows indicate PI(3,4,5)P3 signal. Upper and lower panels are from two biological replicates for each condition. Scale bars: 10 μm. (B) Kinetics of AKT and ERK phosphorylation in HeLa cells after 24 h of SHIP2 inhibition and subsequent H_2_O_2_ treatment (1 mm). Lysate collected and analyzed for the indicated times. Short and long exposure for phosphorylated (p)‐AKT and p‐ERK were shown. (C) HeLa cells were treated with a vehicle or AS19 for 24 h, followed by AKT activator (SC79), 20 µm for 2 h, then 1 mm H_2_O_2_, and then collected at the indicated times. Short and long exposure of the blots were shown. (D) SHIP2 inhibition activates PTEN upon increase in ROS. HeLa cells were treated with vehicle or AS19 for 24 h, then followed by 1 mm H_2_O_2_ for 4 h. Lysates were analyzed for the indicated antibodies. H_2_O_2_ treatment was performed in three biological replicates for each condition. (E) Bar graph summarizing PTEN phosphorylation levels in HeLa cells for each treatment. Data are the means ± SEM from three independent experiments. One‐way ANOVA, *F*(3, 8) = 11.91, *P* = 0.0025. Tukey's multiple comparisons test, **P ˂ 0.01. C‐, vehicle‐treated cells.

The role of SHIP2 as a negative regulator of PI3K/AKT is very well established [19, 23]. For example, upon insulin stimulation, SHIP2 localizes to the plasma membrane to negatively regulate AKT phosphorylation [39]. Contrary to growth factor stimulation [24, 39], fluorescence imaging of GFP–SHIP2 dynamics showed that H_2_O_2_ treatment induces clustering of the protein with minor localization to the plasma membrane. Similar clustering and localization were seen in cells treated with the catalytic inhibitor after H_2_O_2_ treatment (Fig. S3A). It is likely that SHIP2 plays a different function in response to H_2_O_2_. The fact that SHIP2 inhibition induces lower activation of AKT suggests alteration in either PI(3,4,5)P3 synthesis or degradation. To test this hypothesis, I first examined levels of PI(3,4,5)P3 under different conditions. Cells transfected with GFP–PH (pleckstrin homology)–BTK plasmid construct were used. This strategy is based on the use of a specific protein domain, for example, PH–BTK that bind phosphoinositides. When fused to GFP, dynamic changes in phosphoinositides can be visualized *in vitro* [40]. GFP–PH–BTK reporter was used to visualize the PI(3,4,5)P3 in control and SHIP2‐inhibited cells. Consistent with AKT phosphorylation levels, 1 h of H_2_O_2_ stimulation led to marked accumulation of PI(3,4,5)P3 at the plasma membrane in control cells, whereas very low levels of PI(3,4,5)P3 were seen in cells in which SHIP2 was inhibited (Fig. 3A). Consistent with previous findings, PI(3,4,5)P3 accumulation was suppressed upon the PI3K inhibitor, wortmannin [41]. In addition, the kinetics of ERK and AKT activation upon H_2_O_2_ stimulation was dramatically different. H_2_O_2_ treatment leads to marked increase and sustained phosphorylation of both proteins in control cells, whereas only minor phosphorylation of AKT and ERK was seen in SHIP2‐inhibited cells, which then decreased over time (Fig. 3B). Next, I used AKT activator, SC79, a known PI(3,4,5)P3 mimetics that binds the PH domain of AKT and enhances its phosphorylation [42]. As indicated in Fig. 3C, SC79 rescues AKT phosphorylation in cells in which SHIP2 was inactivated. However, this increase occurred only at early time points. At 4 h of H_2_O_2_ treatment, SC79 treatment led to a constant increase in AKT phosphorylation in control cells, whereas no marked change was seen in SHIP2‐inhibited cells (Fig. S3B).

PTEN (phosphatase and tensin homolog) negatively regulates levels of PI(3,4,5)P3 in different contexts and functions as a tumor suppressor by suppressing the AKT signaling pathway [17, 18]. Previous studies have shown that ROS simultaneously activate PI3K and inactivate PTEN via oxidation of cysteine residues within the active site of the protein [43, 44]. Based on our observation that SHIP2 inhibition alters levels of PI(3,4,5)P3 and kinetic activation of AKT, phosphorylation of PTEN at residues Ser380, known as a negative marker of PTEN activity, was analyzed [45]. As indicated in Fig. 3D,E and contrary to vehicle‐treated cells, SHIP2 inhibition markedly enhances PTEN activity as shown by a dramatic decrease in PTEN phosphorylation upon H_2_O_2_ treatment. Together, these experiments demonstrated that SHIP2 inhibition enhances PTEN activation upon H_2_O_2_ treatment, which might negatively affect AKT and ERK phosphorylation.

### PTEN inhibition rescues ΔΨm loss and reduces ROS generation

**Fig. 4:**
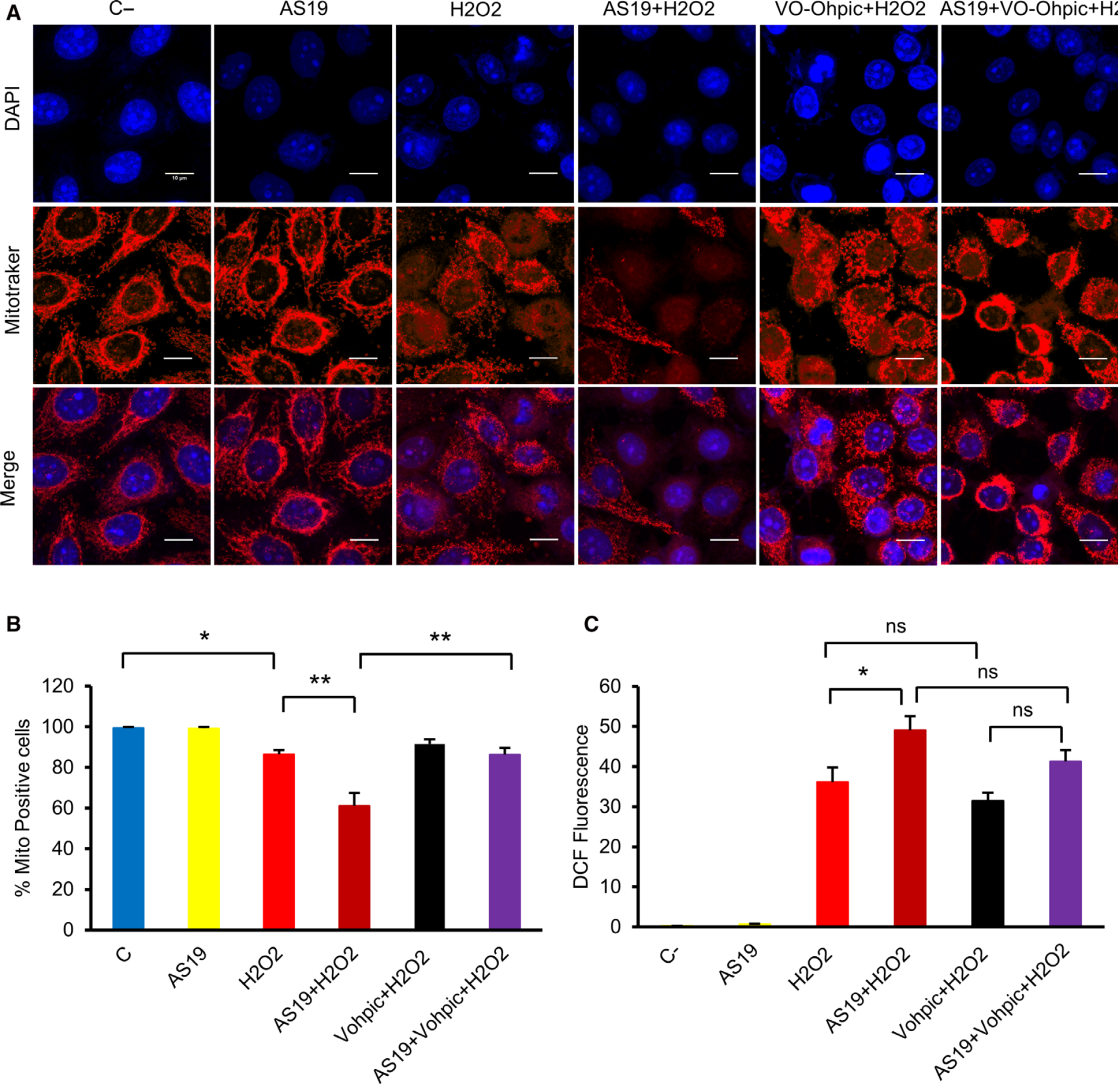
PTEN inhibition partially rescues ΔΨm. (A) HeLa cells were treated with vehicle or AS19 for 24 h, followed by PTEN inhibitor, 2 µm for 2 h, then 1 mm H_2_O_2_ for 3.5 h. Live cells were stained with 100 nm MitoTracker Red for mitochondria (red) during 30 min, then fixed and mounted. Nuclei are stained with DAPI (blue). Scale bars: 10 μm. (B) Bar graph summarizing the percentage of MitoTracker‐positive cells under different conditions. Data are the means ± SEM from three independent experiments. One‐way ANOVA, *F*(5, 73) = 19.54, *P* < 0.0001. (C) PTEN inhibition reduces ROS production. Bar graph summarizing the DCF intensities for each condition. Data are the means ± SEM from three independent experiments. One‐way ANOVA, *F*(5, 174) = 749.2, *P* < 0.0001. Tukey's multiple comparisons test, *P ˂ 0.05, **P ˂ 0.01. C‐, vehicle‐treated cells; ns, not significant.

Previous findings have shown that ROS trigger localization of PTEN to mitochondria [46]. Further, it has been reported that a fraction of PTEN localizes to the endoplasmic reticulum (ER) and mitochondria‐associated membranes to modulate intracellular calcium (Ca^2+^) release from the ER to mitochondria in response to apoptotic stimuli [47]. Therefore, I examined the effect of simultaneous inhibition of SHIP2 and PTEN on mitochondrial properties. The percentage of cells displaying positive staining for MitoTracker Red was measured. As shown in Fig. 4A,B, PTEN inhibition led to a significant increase in proportion of intact mitochondria specifically in cells in which SHIP2 was inactivated. PTEN inhibition affected not only ΔΨm but also mitochondria morphology. Simultaneous inhibition of PTEN and SHIP2 induced perinuclear aggregate of hyperfused mitochondria. Although not examined in this study, it should be noted that two very recent studies reported that the long isoform of PTEN plays a role in mitophagy, a process that involves selective degradation of mitochondria by autophagy [48, 49]. In addition, PTEN inhibition moderately decreased ROS production upon H_2_O_2_ treatment in cells in which SHIP2 was blocked (Fig. 4C).

### PTEN inhibition rescues ERK activation

**Fig. 5:**
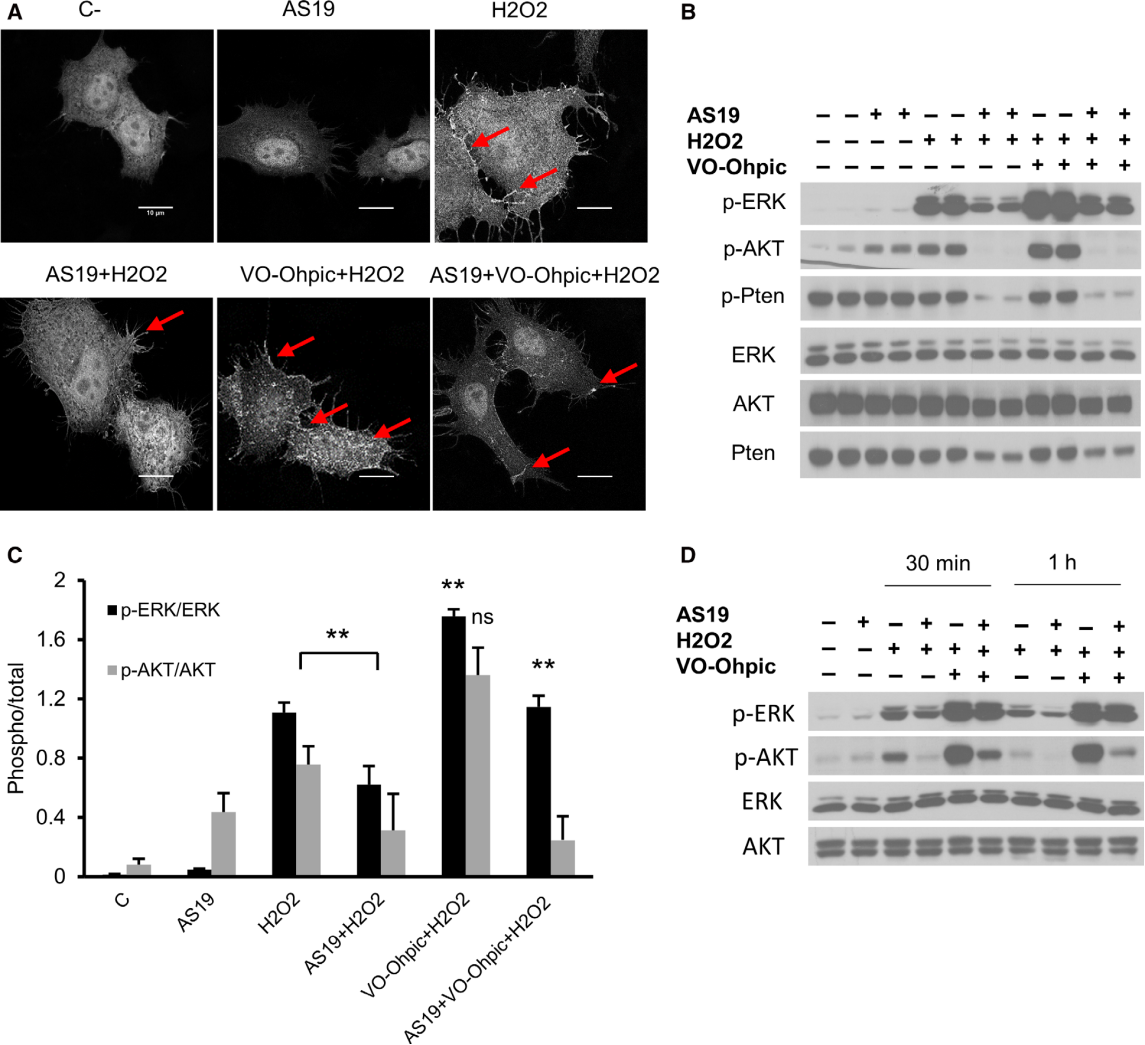
Catalytic inhibition of PTEN rescues PI(3,4)P2 levels and ERK phosphorylation. (A) HeLa cells were first transfected with GFP‐tagged TAPP1‐PH domain plasmid; 6 h later they were treated overnight with vehicle or AS19 followed by 1 mm H_2_O_2_ for 1 h. Cells were then fixed and mounted. Fluorescent images of PI(3,4)P2 levels are shown. Cells pretreated with 2 µm PTEN inhibitor (VO‐OHpic) were used as a control. Arrows indicate PI(3,4)P2 signal. Scale bars: 10 μm. (B) PTEN inhibition rescues MAPK activation. HeLa cells were treated with vehicle or AS19 for 24 h, followed by 2 µm PTEN inhibitor for 2 h, then 1 mm H_2_O_2_ for 4 h. Cell lysates were analyzed for the indicated antibodies. (C) Bar graph summarizing ERK and AKT phosphorylation levels for each treatment. Data are the means ± SEM from three independent experiments, each in duplicates. p‐ERK/ERK, one‐way ANOVA, *F*(5, 12) = 101.1, *P* < 0.0001. Tukey's multiple comparisons test, **P ˂ 0.01. p‐AKT/AKT, one‐way ANOVA, *F*(5, 12) = 8.468. (D) Catalytic inhibition of PTEN partially rescues AKT phosphorylation only at the early time point. HeLa cells were treated as in (B), then collected at the indicated times. C‐, vehicle‐treated cells.

To mechanistically understand the impact of PTEN overactivation induced by SHIP2 inhibition and ROS accumulation, I first examined the lipid phosphatase activity of PTEN. Given that PI(3,4)P2 is also known as a major substrate for PTEN and is required for full activation of AKT [50, 51], I used the GFP–PH–TAPP1 reporter to visualize PI(3,4)P2 in control and SHIP2‐inactivated cells. One hour of H_2_O_2_ treatment led to marked cellular accumulation of PI(3,4)P2 in control cells, whereas very low levels of PI(3,4)P2 were seen in cells in which SHIP2 was inhibited (Fig. 5A). Interestingly, PTEN inhibition and H_2_O_2_ treatment led to a substantial increase of PI(3,4)P2 in control cells. However, only moderate accumulation of PI(3,4)P2 was seen in cells treated with SHIP2 and PTEN inhibitors after H_2_O_2_ treatments. Next, I analyzed the effect of change in PTEN activity on ERK and AKT phosphorylation upon 4 h of H_2_O_2_ treatment. Western blot analysis showed that pretreatment of control cells with PTEN inhibitor significantly increased phosphorylation of both ERK and AKT. Surprisingly, in SHIP2‐inactivated cells, PTEN inhibition had only a minor effect on AKT phosphorylation but rather rescued MAPK activation, as indicated by a significant increase in ERK phosphorylation (Fig. 5B,C). To further ascertain that PTEN inhibition has only a minor effect on AKT phosphorylation in SHIP2‐inhibited cells, I analyzed kinetic activation of ERK and AKT upon simultaneous H_2_O_2_ treatment and PTEN inhibition at earlier time points. PTEN inhibition led to a substantial increase in ERK phosphorylation in all conditions. However, when compared with control cells, an increase in AKT phosphorylation upon simultaneous SHIP2 and PTEN inhibition was seen only at 30 min after H_2_O_2_ treatment (Fig. 5D). I conclude that SHIP2 inhibition induces constitutive activation of PTEN upon the increase of ROS, and that both lipid and protein phosphatase of PTEN might be affected.

### SHIP2 inhibition alters both lipid and protein phosphatase activity of PTEN

**Fig. 6:**
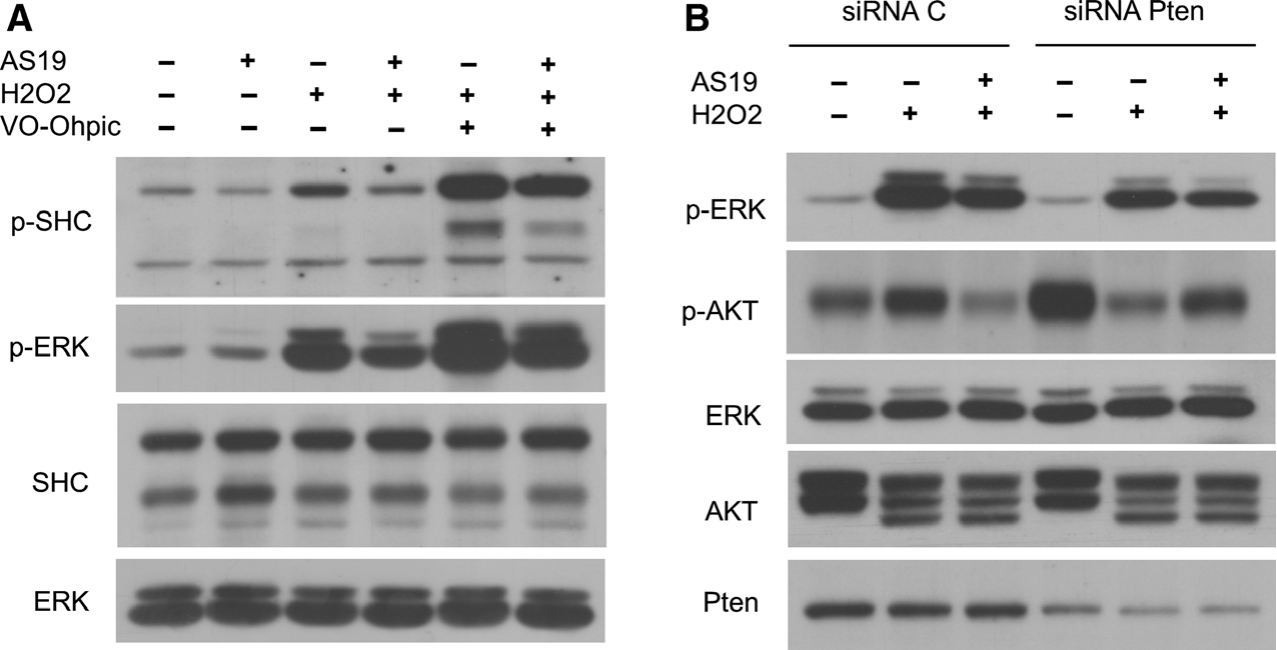
SHIP2 inhibition alters both lipid and protein phosphatase activity of PTEN. (A) PTEN inhibition activates SHC. HeLa cells were treated with vehicle or AS19 for 24 h followed by PTEN inhibitor (2 µm) during 2 h and 1 mm H_2_O_2_ for 4 h. Lysate was blotted for the indicated antibodies. Representative image of two independent experiments. (B) PTEN knockdown rescues AKT phosphorylation. HeLa cells were transfected with siRNA control or siRNA directed to PTEN. Twenty‐four hours later, they were treated with vehicle or SHIP2 inhibitor for 24 h, then followed by 1 mm H_2_O_2_ during 4 h. Lysate was analyzed for the indicated antibodies. Representative image of two independent experiments.

The earlier results showed that chemical inhibition of PTEN rescued only MAPK activation and ΔΨm, suggesting that overactivation of PTEN seen upon SHIP2 inhibition could also involve its protein phosphatase activity. Indeed, in addition to its lipid phosphatase activity, previous reports have shown that PTEN is able to dephosphorylate the isoform p52Shc of Src homology 2 domain‐containing protein (SHC)‐transforming protein 1, leading to down‐regulation of the Ras–ERK pathway activity [52, 53]. Therefore, I examined p52Shc phosphorylation dynamics under different conditions. As indicated in Fig. 6A, SHIP2 inhibition reduces both basal and H_2_O_2_‐induced p52Shc phosphorylation. Interestingly, PTEN inhibition increased phosphorylation of both ERK and p52Shc, further indicating that SHIP2 inhibition enhances both lipid and protein phosphatase activity of PTEN upon H_2_O_2_ treatment. These findings were further validated by siRNA‐mediated knockdown of PTEN. Indeed, reduced PTEN protein level induces lower ERK phosphorylation in both conditions while rescuing AKT phosphorylation in SHIP2‐inhibited cells (Fig. 6B). Altogether, these findings demonstrated that in cervical cancer cell lines, SHIP2 inhibition and H_2_O_2_‐induced ROS generation enhance both lipid and protein phosphatase activity of PTEN, which, in turn, down‐regulates MAPK and PI3K/Akt pathways.

## Discussion

ROS play a crucial role during organismal development. At lower concentrations, they are involved in activating diverse signaling pathways, leading to cell growth and differentiation. At high levels, or dysregulation in cellular redox balances, ROS induce oxidative stress [1, 2]. Dysregulation in redox balance has been implicated in various pathophysiological conditions, including cancer, neurodegeneration and alteration in the immune system [54]. Oxidative stress induces inflammation and causes cell death by apoptosis or necrosis [6, 7]. Recent studies have highlighted the role of SHIP2 in modulating cellular signaling pathways in response to different stressors. As a negative regulator of PI(3,4,5)P3 level, SHIP2 inhibition in liver cancer cells enhances PI3K/AKT signaling and prevents ROS production after palmitate treatment [29], whereas its overexpression suppresses PI3K/AKT signaling and cell growth in gastric cancer cells [27]. SHIP2 and PTEN are the major negative regulators of PI3K/AKT signaling [18, 19, 20], and both proteins are highly expressed in cervical cancer cell lines HeLa and SiHa (Fig. 1A and Fig. S1). In this study, I provide the first evidence that SHIP2 inhibition in these cells induces PTEN activation upon H_2_O_2_ treatment, which, in turn, alters MAPK and PI3K/AKT signaling pathways. Moreover, and contrary to previous findings in liver cancer cell lines [29], SHIP2 inhibition in cervical cancer cell lines enhances ROS production, a common consequence of oxidative stress‐mediated cell death. In this regard, ROS scavenger pyruvate reactivated PI3K/AKT signaling and prevented H_2_O_2_‐induced cell death (Fig. 2B–D).

Our data suggest that SHIP2 plays a different function in cell‐type‐ and stimuli‐dependent manners. Indeed, growth factor stimulation enhances AKT phosphorylation, whereas H_2_O_2_ attenuates AKT phosphorylation upon SHIP2 inactivation (Fig. 1B,C). The role of SHIP2 as a lipid phosphatase in response to growth factor is very well established. It accumulates at the plasma membrane and modulates PI3K downstream signaling through its lipid phosphatase activity [23, 39], whereas H_2_O_2_ treatment induces clustering of the protein with minor localization to the plasma membrane (Fig. S3A), demonstrating stimuli‐dependent dynamics of SHIP2.

Together, our results, at least in cervical cancer cells, establish a concept that in response to growth factors, SHIP2 localizes to the plasma membrane and prevents sustained activation of both PI3K/Akt and MAPK through its lipid phosphatase activity; however, in response to H_2_O_2_, it is likely that SHIP2 plays a role as a scaffolding protein to maintain sustained activation of PI3K/Akt and MAPK. Consistently, lipid phosphate‐independent roles of SHIP2 in signal transduction have been reported recently. Indeed, in human embryonic kidney cells, HEK293T, loss of SHIP2 alters ERK activation in response to fibroblast growth factor stimulation [55]. In accordance with these findings, our observations suggest that the inhibitor might not only block the catalytic activity of SHIP2 but also its scaffolding function by altering SHIP2 interaction with its partners. Therefore, a comprehensive study would be needed to clarify how this chemical inhibitor affects SHIP2 structural conformation and its interaction with other proteins.

One of the most interesting findings in this study is that SHIP2 inhibition induces overactivation of PTEN upon H_2_O_2_ treatment, which, in turn, lowers the levels of PI(3,4,5)P3 and PI(3,4)P2 and suppresses AKT activation. Surprisingly, although chemical inhibition of PTEN rescued ERK phosphorylation, no marked change in AKT phosphorylation was seen (Fig. 5B,C). These results suggest activation of both lipid and protein phosphates of PTEN upon SHIP2 inhibition. Indeed, in addition to its lipid phosphatase, PTEN possesses a protein phosphatase activity. For example, Gu *et al*. [53] demonstrated that PTEN dephosphorylates SHC and suppresses ERK activation. Consistent with these findings, PTEN inhibition enhances p52Shc phosphorylation (Fig. 6A). To further examine the role of PTEN protein upon SHIP2 inhibition, I used siRNA knockdown. I could provide evidence that down‐regulation of PTEN rescued AKT phosphorylation upon SHIP2 inhibition (Fig. 6B). Notably, PTEN was not fully abolished by siRNA, and the remaining protein might be sufficient to block overactivation of AKT upon SHIP2 inhibition. Altogether the results presented here highlight crosstalk between SHIP2 and PTEN. The mechanistic crosstalk was not examined. However, two possibilities might explain PTEN overactivation. One possibility is that an increase in PI(4,5)P2 upon SHIP2 inhibition (Fig. S4) might enhance PTEN phosphatase activity. Indeed, PI(4,5)P2 is known as a cofactor that binds PTEN and enhances its lipid phosphatase activity [56]. Alternatively, or in addition to the first possibility, structural changes of SHIP2 upon binding to AS19 release SHC, which then can be dephosphorylated by PTEN. Indeed, SHIP2 and PTEN also modulate MAP and PI3K activity upon binding to SHC [39, 53]. The signaling mechanism that drives PTEN overactivation upon SHIP2 inhibition is beyond the scope of this study, but in future studies, it would be important to reveal the mechanistic crosstalk between these two proteins, particularly in cancer development. Indeed, SHIP2 is involved in the regulation of several cellular processes, such as adhesion and migration, which are known hallmarks of cancer progression and metastasis [24, 25, 26]. In this regard, Hoekstra *et al*. [57] reported that SHIP2 is highly expressed in colorectal cancer tissue. Consistent with our observations, SHIP2 inhibition in colorectal cancer cells reduces AKT phosphorylation [57]. Altogether, these findings suggest that SHIP2 might play a role in cancer development and progression, providing a new potential therapeutic target.

## Materials and methods

### Reagents

Dulbecco's modified Eagle's medium (DMEM) was purchased from Gibco [Catalog (Cat.) No. 41966052] (Fisher Scientific), SHIP2 inhibitor AS19 from Sigma (Cat. No. SML1022), H_2_O_2_ from Sigma, PTEN inhibitor VO‐Ohpic trihydrate from Selleckchem (Cat. No. S8174), 2′,7′‐DCFDA from Sigma (Cat. No D6883), 3‐methyladenine from Sigma (Cat. No. M9281), MitoTracker Red CMXRos from Cell Signaling (Cat. No. 9082), and 4′,6‐diamidino‐2‐phenylindole dihydrochloride (DAPI) from Molecular Probes (Life Technologies, Carlsbad, CA, USA).

### Cell culture

HEK293T and HeLa cells were obtained from S. Schurmans (GIGA‐Laboratory of Functional Genetics, University of Liege). SiHa cells were obtained from S. Kateryna (GIGA‐University of Liege).

Cells were grown as recommended in DMEM containing 4.5 g·L^−1^ d‐glucose supplemented with 10% FBS, 100 U·mL^−1^ penicillin, 100 U·mL^−1^ streptomycin and 1% l‐glutamine (Gibco). Cells between 3 and 10 passages were used. All cultures were maintained at 37 °C in a humidified 5% CO_2_ incubator. Unless otherwise stated, each cell was treated with 10 µm SHIP2 inhibitor AS1949490 or equivalent volume of DMSO as a control for 24 h.

### Determination of ROS levels

Cytoplasmic ROS levels were measured using the fluorescent probe 2′,7′‐DCFDA in a final concentration of 25 μm. Cells were seeded overnight at equal density, 15 000 cells per well in a 96‐well plate. Upon 24 h treatment with DMSO as vehicle or SHIP2 inhibitor (10 μm), DCFDA was supplemented to the growth medium and incubated for 30 min at 37 °C. Stained cells were rinsed with warm basal DMEM and incubated again for 1 h in fresh medium. Where indicated, pyruvate pretreatment (5 mm) for 1 h was followed by H_2_O_2_ (1 mm). Fluorescent signal detection was measured using Tecan Infinite M200 PRO Multi‐Detection Microplate Reader (Tecan Life Science) or VICTOR™ X Series Multilabel Plate Readers (PerkinElmer) set to 485‐nm excitation and 530‐nm emission wavelength.

### Immunofluorescence

Cells were plated overnight on glass coverslips in 24‐well plates (150 000 cells/well) followed by treatment with vehicle or SHIP2 inhibitor for 24 h. For mitochondria staining, live cells were supplemented with 100 nm MitoTracker Red for 30 min at 37 °C. With different treatments, cells were fixed using ice‐cold methanol at −20 °C during 10 min. After three washes with PBS, cells were then incubated with DAPI (0.1 µg·mL^−1^). Images were captured using Leica TCS SP5 microscope and processed with imagej (National Institutes of Health, Bethesda, MD, USA).

### siRNA transfections

siRNA transfection was performed using Dharmafect 4 according to the manufacturer's instruction (Dharmacon). In brief, cells were plated a day before at 50% confluence. They were then transfected with 75 nm siRNA. Twenty‐four hours later, the medium was replaced, then treated with vehicle or SHIP2 inhibitor for 24 h where indicated. The following siRNAs were used, ON‐TARGET plus SMART pool siRNA against PTEN (#L003023‐00‐0005) and a control siRNA (#D‐001810‐10‐05).

### Plasmid transfection

Plasmid transfection was performed using Lipofectamine 3000 according to the manufacturer's instructions. The following plasmid constructs GFP–SHIP2, GFP–BTK–PH, GFP–TAPP1–PH and GFP–phospholipase C, delta (PLCδ)–PH were obtained from S. Schurmans (GIGA‐Laboratory of Functional Genetics, University of Liege).

### Western blotting

Cells were first washed with PBS and then resuspended in radioimmunoprecipitation assay buffer (150 mm NaCl, 1.0% Nonidet P‐40, 0.5% sodium deoxycholate, 0.1% SDS, 50 mm Tris–HCl, pH 8.0), containing protease and phosphatase inhibitors (ROCHE). Protein samples were collected by centrifugation (10 000 ***g***, 10 min) at 4 °C. Sample concentrations were quantified using Bio‐Rad Protein Assay according to the manufacturer's instructions. Equal amounts of protein extract were loaded and separated by SDS/PAGE and then transferred to nitrocellulose membranes. The membranes were blocked for 60 min at room temperature with 5% BSA in Tris‐buffered saline supplemented with 0.05% Tween 20. Membranes were then incubated with primary antibodies overnight at 4 °C, followed by horseradish peroxidase‐labeled secondary antibodies. Labeled proteins were visualized with an enhanced chemiluminescence system (Fisher Scientific). Optical densities of proteins were determined using imagej.

The following antibodies were all purchased from Cell Signaling [phospho‐AKT S473 #4060, AKT #4691, phospho‐ERK (Thr202/Tyr204) #4377, ERK #4695, PARP #9542, Phospho‐MEK1/2 (Ser217/221) #9121, MEK1/2 #9122, phosphor‐Pten S380 #9551, Pten #5959, vinculin #13901 and phospho‐SHC (Tyr239/240) #2434]. Horseradish peroxidase‐linked secondary antibodies, Rabbit A0545 and Mouse A9044, were from Sigma. Actin and SHC were from Santa Cruz Biotechnology (SC‐1616 and SC‐967, respectively). Anti‐SHIP2 Ig was a gift from C. Erneux (IRIBHM, Université Libre de Bruxelles, Belgium).

### Cell viability

Cell viability was measured using Cell Counting Kit‐8 (CCK‐8) (Cat. No. 96992; Sigma). Cells were first seeded overnight at equal density, 15 000 cells per well in a 96‐well plate. They were then treated with vehicle or SHIP2 inhibitor for 24 h followed by 1 mm H_2_O_2_ for 4 h and treated with WST‐8 for 1 h at 37 °C. The absorbance at 450 nm (*A*_450 nm_) was then measured using Tecan Infinite M200 PRO Multi‐Detection Microplate Reader.

### ΔΨm measurement

ΔΨm measurement was performed using Cell Meter™ Mitochondrion Membrane Potential Assay Kit (AAT Bioquest #22805). HeLa cells were seeded overnight at equal density, 10 000 cells per well in a 96‐well plate, then treated with vehicle or 10 µm SHIP2 inhibitor for 24 h. Upon 1 mm H_2_O_2_ treatment during 90 min, MitoTell™ Orange solution was added and incubated for 30 min at 37 °C. Assay buffer B was then added. Fluorescence was then measured using Infinite M200 PRO Multi‐Detection Microplate Reader. Excitation wavelength was set at 540 nm and emission wavelength at 590 nm. Quantification was performed relative to control untreated cells. HeLa cells treated with carbonyl cyanide *m*‐chlorophenyl hydrazone 20 µm (ab141229; Abcam) were used as a control.

### Statistical analysis

Statistical analyses were performed using graphpad prism 7 Software (GraphPad Software, San Diego, CA, USA). Data were presented as mean ± SD. For comparisons of three or more conditions, one‐way ANOVA with Tukey's honestly significant difference *post hoc* test was used. Differences less than 0.05 were considered statistically significant.

## Conflict of interest

The author declares no conflict of interest.

## Author contributions

AA conceived and designed the experiments; acquired, analyzed, and interpreted the data; and wrote the manuscript.

## Supporting information


**Fig. S1.** (A, B) RNA levels of SHIP2 and PTEN in different cell lines [30,31]. See also The Human Protein Atlas (https://www.proteinatlas.org/ENSG00000165458‐INPPL1/cell and https://www.proteinatlas.org/ENSG00000171862‐PTEN/cell).
**Fig. S2.** SHIP2 inhibition alters AKT activation upon H_2_O_2_ treatment in SiHa cells. (A) SiHa cells were first treated with vehicle or SHIP2 inhibitor for 48 h, followed by 1 mM H_2_O_2_ for 6 h. Cell lysates were analyzed for the indicated antibodies. H_2_O_2_ treatment was performed in biological triplicates for vehicle and SHIP2‐inhibited cells. (B) The ROS scavenger pyruvate prevents H_2_O_2_‐induced cell death. SiHa cells were first treated with vehicle or SHIP2 inhibitor for 48 h, followed by pyruvate (5 mM) for 2 h, and then treated with 1 mM H_2_O_2_ for 4 h. Cell lysates were analyzed for the indicated antibodies. (C) SiHa cells were treated as in (B); then cell viability was measured with CCK‐8 assays. After 5 h of 1 mM H_2_O_2_, cells were treated with WST‐8 for 1 h at 37 °C. The *A*_450 nm_ was measured with a microplate reader. Data are the means ± SEM from two independent experiments, each in four replicates. One‐way ANOVA, *F*(5, 62) = 92.1, *P* < 0.0001. Tukey's multiple comparisons test, **P ˂ 0.01.
**Fig. S3.** H_2_O_2_ induces SHIP2 clusters formation. (A) HeLa cells were first transfected with GFP plasmid alone as a control or with GFP–SHIP2. Twenty‐four hours later, cells were treated with vehicle or SHIP2 inhibitor for 24 h, then followed by 1 mM H_2_O_2_ for 1 h. Cells were then fixed and mounted. Scale bar: 10 μM. (B) HeLa cells were treated with a vehicle or AS19 for 24 h and subsequently followed by AKT activator (SC79), 20 µM for 2 h and 1 mM H_2_O_2_ for 4 h. Total lysates were analyzed for the indicated antibodies.
**Fig. S4.** SHIP2 inhibition enhances PI(4,5)P2 accumulation. HeLa cells were first transfected with plasmid GFP‐tagged PLCδ‐PH domain; 6 h later they were treated with vehicle or AS19 for 24 h, then fixed and mounted. Fluorescent images of PI(4,5)P2 levels are shown. Scale bar: 10 μM. C‐, vehicle‐treated cells.

**Appendix S1.** Original uncropped western blot figures. Some pictures are with additional samples data. However, these samples are not related to the results of this article (please see where indicated).

## Data Availability

The raw data of this study are available from the corresponding author on reasonable request.
