# Supplementary material for: SHIP2 inhibition alters redox‐induced PI3K/AKT and MAP kinase pathways via PTEN over‐activation in cervical cancer cells
Source: FEBS Open Bio. 2020 Oct 1;10(10):2191–205. doi: 10.1002/2211-5463.12967 (PMC7530381; doi:10.1002/2211-5463.12967)

Figure S1

THE HUMAN PROTEIN ATLAS

a **Cell lines sorted on descending RNA expression of SHIP2.**

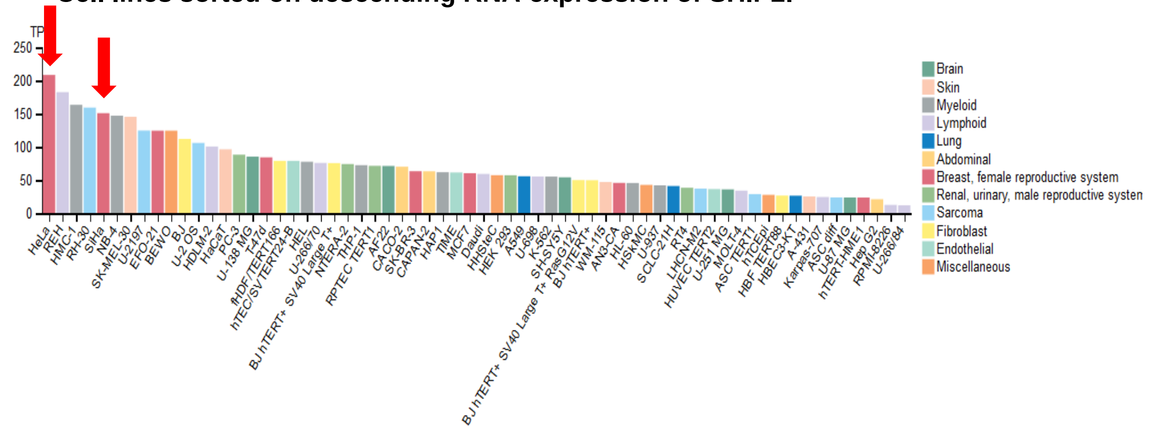

b **Cell lines sorted on descending RNA expression of Pten.**

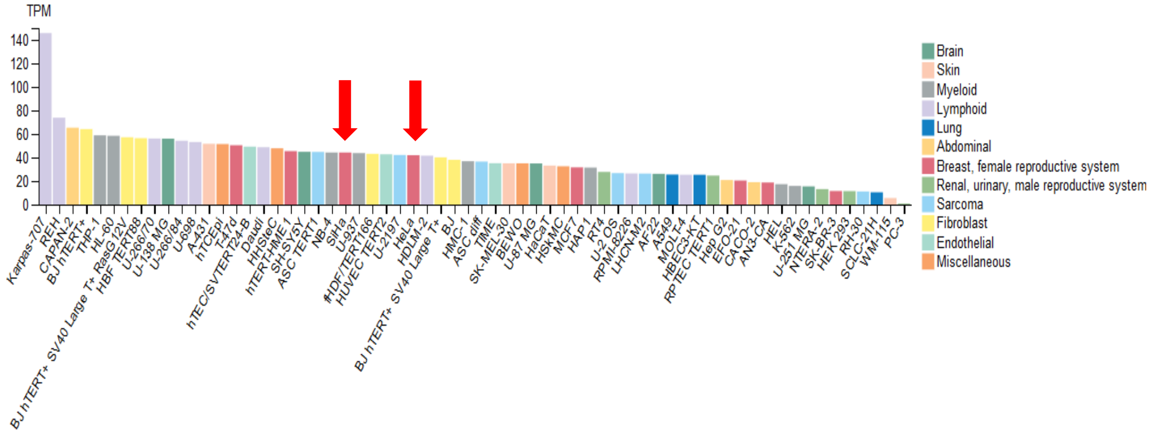

image/gene/data available from

https://www.proteinatlas.org/ENSG00000165458-INPPL1/cell

https://www.proteinatlas.org/ENSG00000171862-PTEN/cell

Figure S2

Siha Cells

a

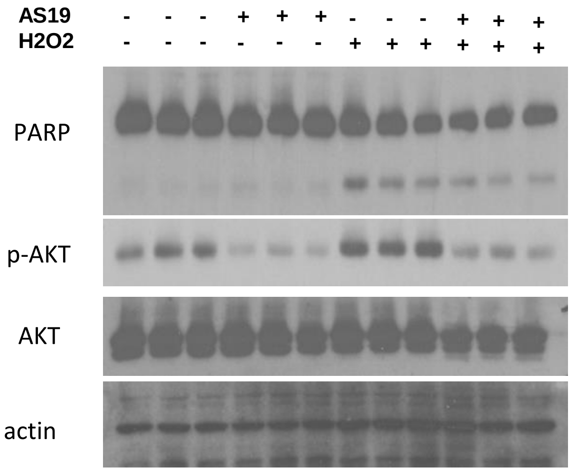

b

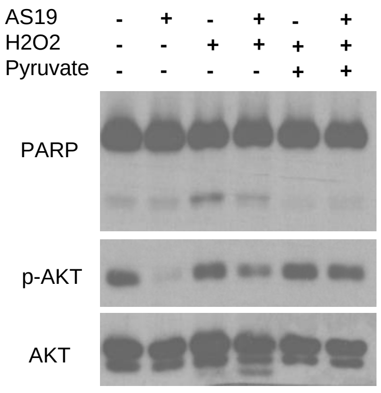

c

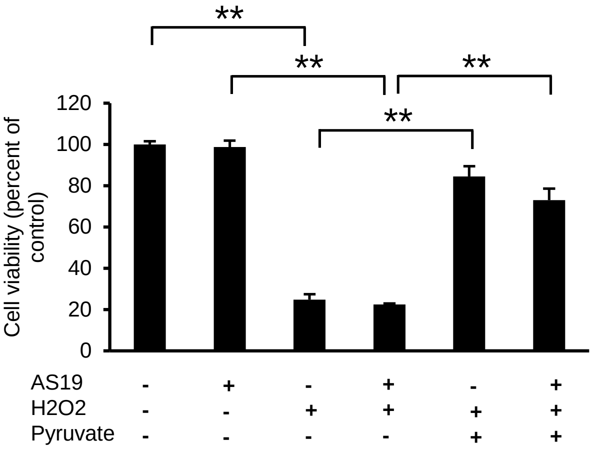

Figure S3

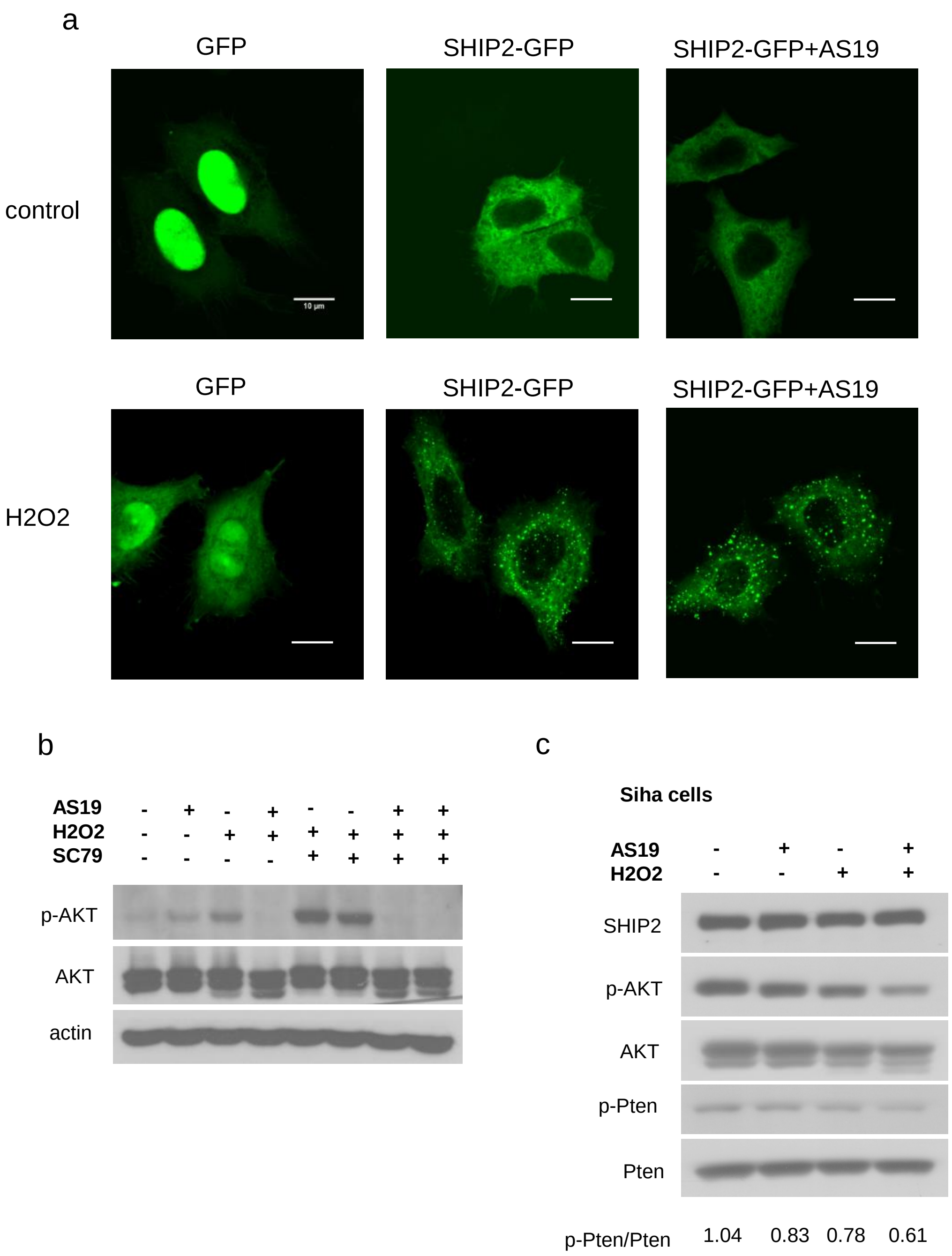
a
GFP
SHIP2-GFP
SHIP2-GFP+AS19
control
10 μm
GFP
SHIP2-GFP
SHIP2-GFP+AS19
H2O2
b
AS19 - + - + - - + +
H2O2 - - + + + + + +
SC79 - - - - + + + +
p-AKT
AKT
actin
c
Siha cells
AS19 - + - +
H2O2 - - + +
SHIP2
p-AKT
AKT
p-Pten
Pten
p-Pten/Pten 1.04 0.83 0.78 0.61

Figure S4

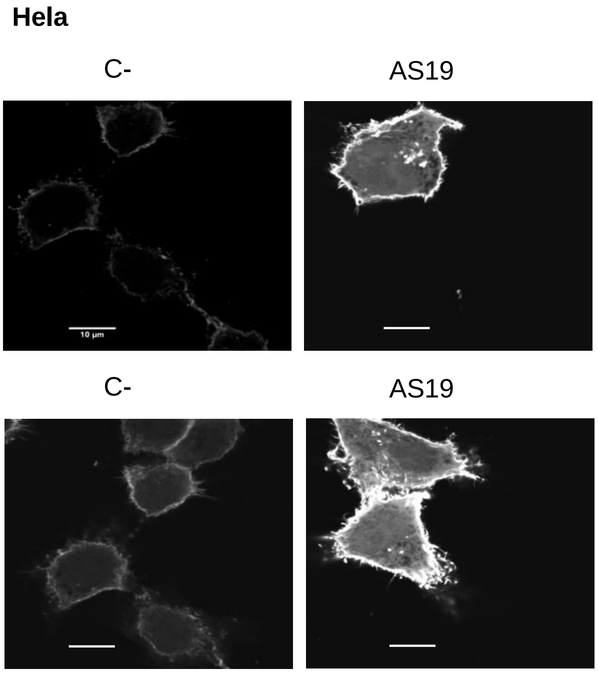

Supplement: Supplementary file 1 — Fig. S1. (A, B) RNA levels of SHIP2 and PTEN in different cell lines [30,31]. See also The Human Protein Atlas (https://www.proteinatlas.org/ENSG00000165458‐INPPL1/cell and https://www.proteinatlas.org/ENSG00000171862‐PTEN/cell). Fig. S2. SHIP2 inhibition alters AKT activation upon H2O2 treatment in SiHa cells. (A) SiHa cells were first treated with vehicle or SHIP2 inhibitor for 48 h, followed by 1 mM H2O2 for 6 h. Cell lysates were analyzed for the indicated antibodies. H2O2 treatment was performed in biological triplicates for vehicle and SHIP2‐inhibited cells. (B) The ROS scavenger pyruvate prevents H2O2‐induced cell death. SiHa cells were first treated with vehicle or SHIP2 inhibitor for 48 h, followed by pyruvate (5 mM) for 2 h, and then treated with 1 mM H2O2 for 4 h. Cell lysates were analyzed for the indicated antibodies. (C) SiHa cells were treated as in (B); then cell viability was measured with CCK‐8 assays. After 5 h of 1 mM H2O2, cells were treated with WST‐8 for 1 h at 37 °C. The A 450 nm was measured with a microplate reader. Data are the means ± SEM from two independent experiments, each in four replicates. One‐way ANOVA, F(5, 62) = 92.1, P < 0.0001. Tukey's multiple comparisons test, **P ˂ 0.01. Fig. S3. H2O2 induces SHIP2 clusters formation. (A) HeLa cells were first transfected with GFP plasmid alone as a control or with GFP–SHIP2. Twenty‐four hours later, cells were treated with vehicle or SHIP2 inhibitor for 24 h, then followed by 1 mM H2O2 for 1 h. Cells were then fixed and mounted. Scale bar: 10 μM. (B) HeLa cells were treated with a vehicle or AS19 for 24 h and subsequently followed by AKT activator (SC79), 20 µM for 2 h and 1 mM H2O2 for 4 h. Total lysates were analyzed for the indicated antibodies. Fig. S4. SHIP2 inhibition enhances PI(4,5)P2 accumulation. HeLa cells were first transfected with plasmid GFP‐tagged PLCδ‐PH domain; 6 h later they were treated with vehicle or AS19 for 24 h, then fixed and mounted. Fluorescent images of PI(4,5) [file FEB4-10-2191-s001.pdf]
