# Supplementary material for: SHIP2 inhibition alters redox‐induced PI3K/AKT and MAP kinase pathways via PTEN over‐activation in cervical cancer cells
Source: FEBS Open Bio. 2020 Oct 1;10(10):2191–205. doi: 10.1002/2211-5463.12967 (PMC7530381; doi:10.1002/2211-5463.12967)
Supplement: Supplementary file 2 — Appendix S1. Original uncropped western blot figures. Some pictures are with additional samples data. However, these samples are not related to the results of this article (please see where indicated). [file FEB4-10-2191-s002.pptx]

## Slide 1
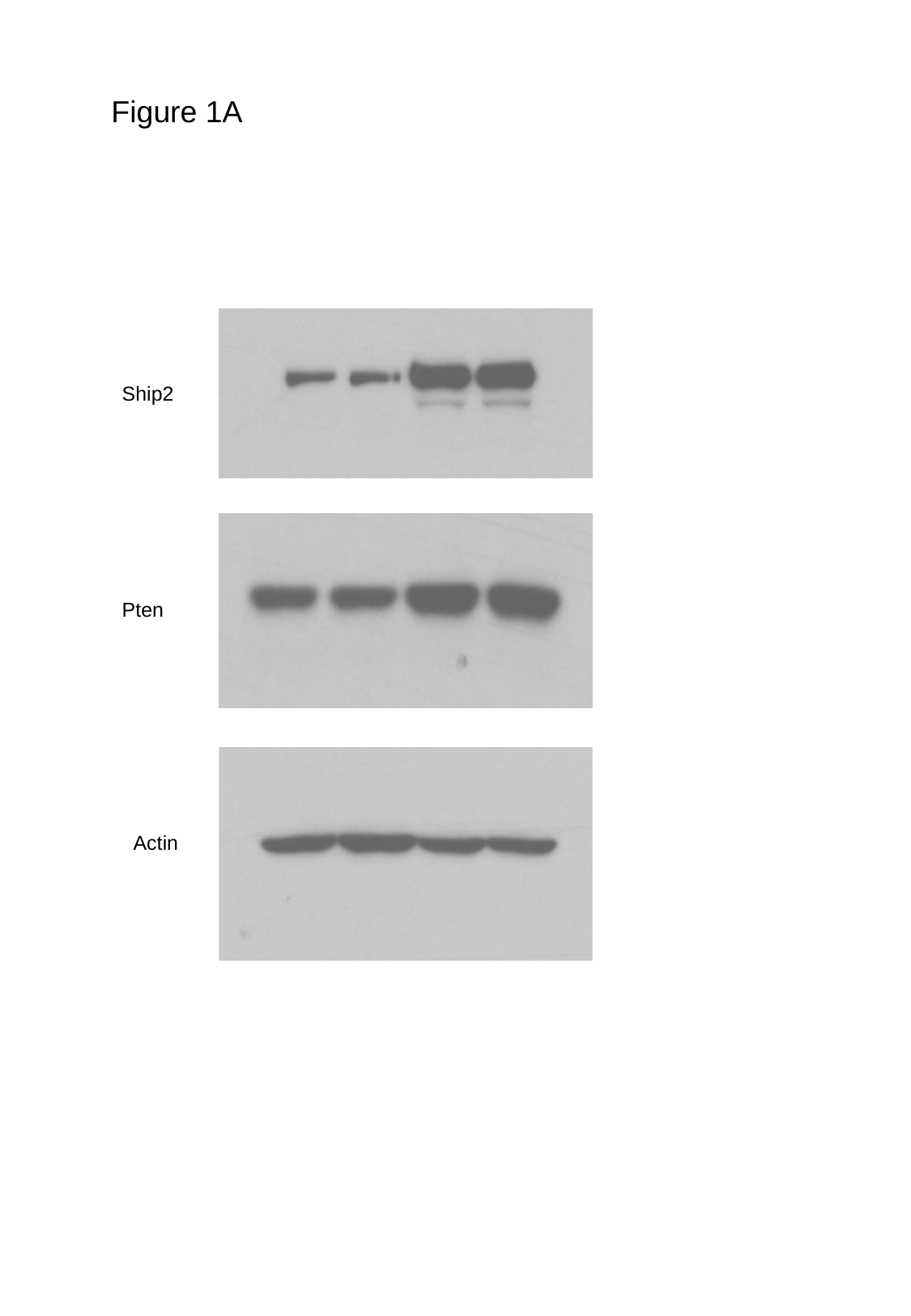

Figure 1A
Ship2
Pten
Actin

## Slide 2
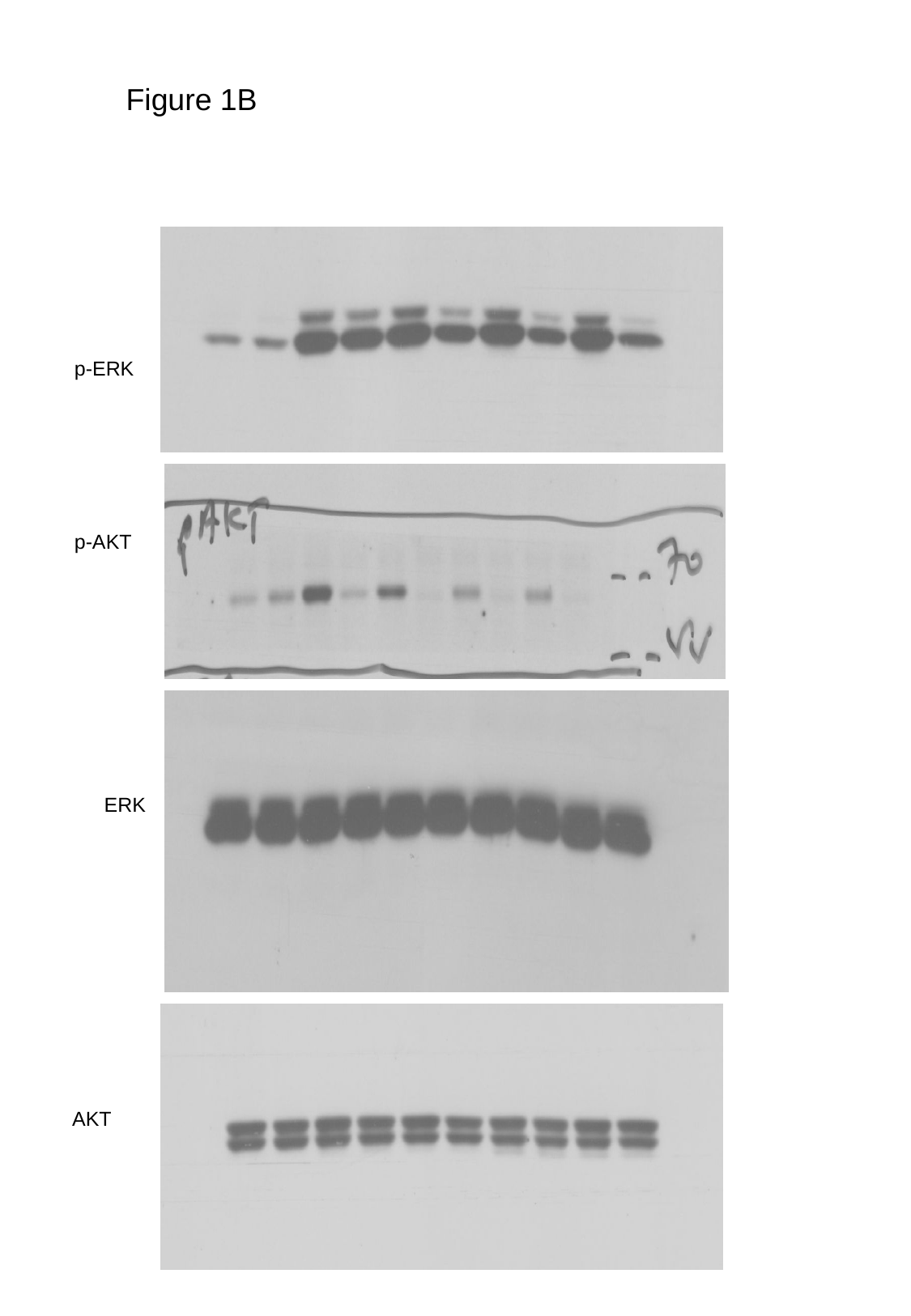

Figure 1B
p-ERK
p-AKT
ERK
AKT

## Slide 3
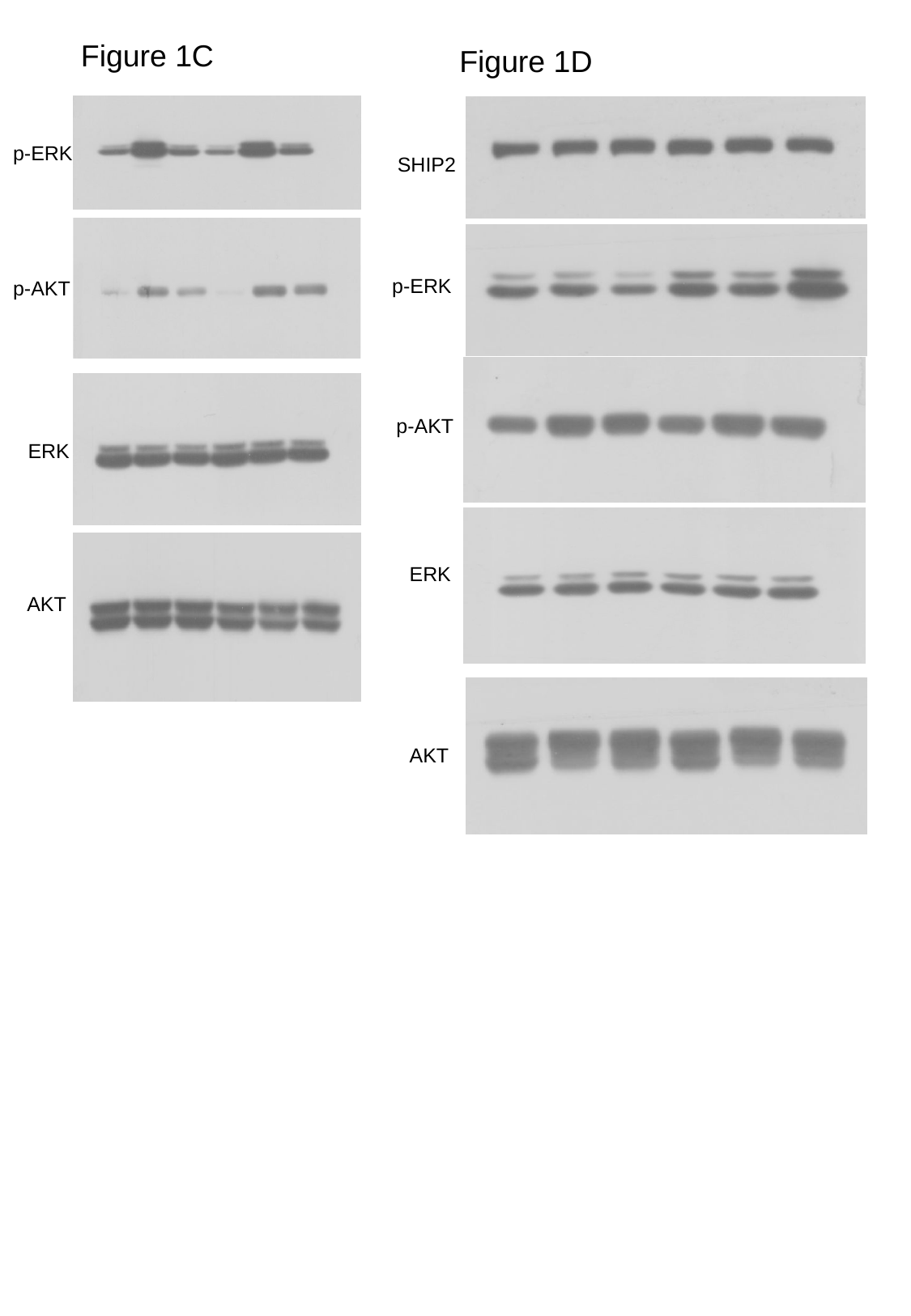

Figure 1C
Figure 1D
p-ERK
SHIP2
p-ERK
p-AKT
p-AKT
ERK
ERK
AKT
AKT

## Slide 4
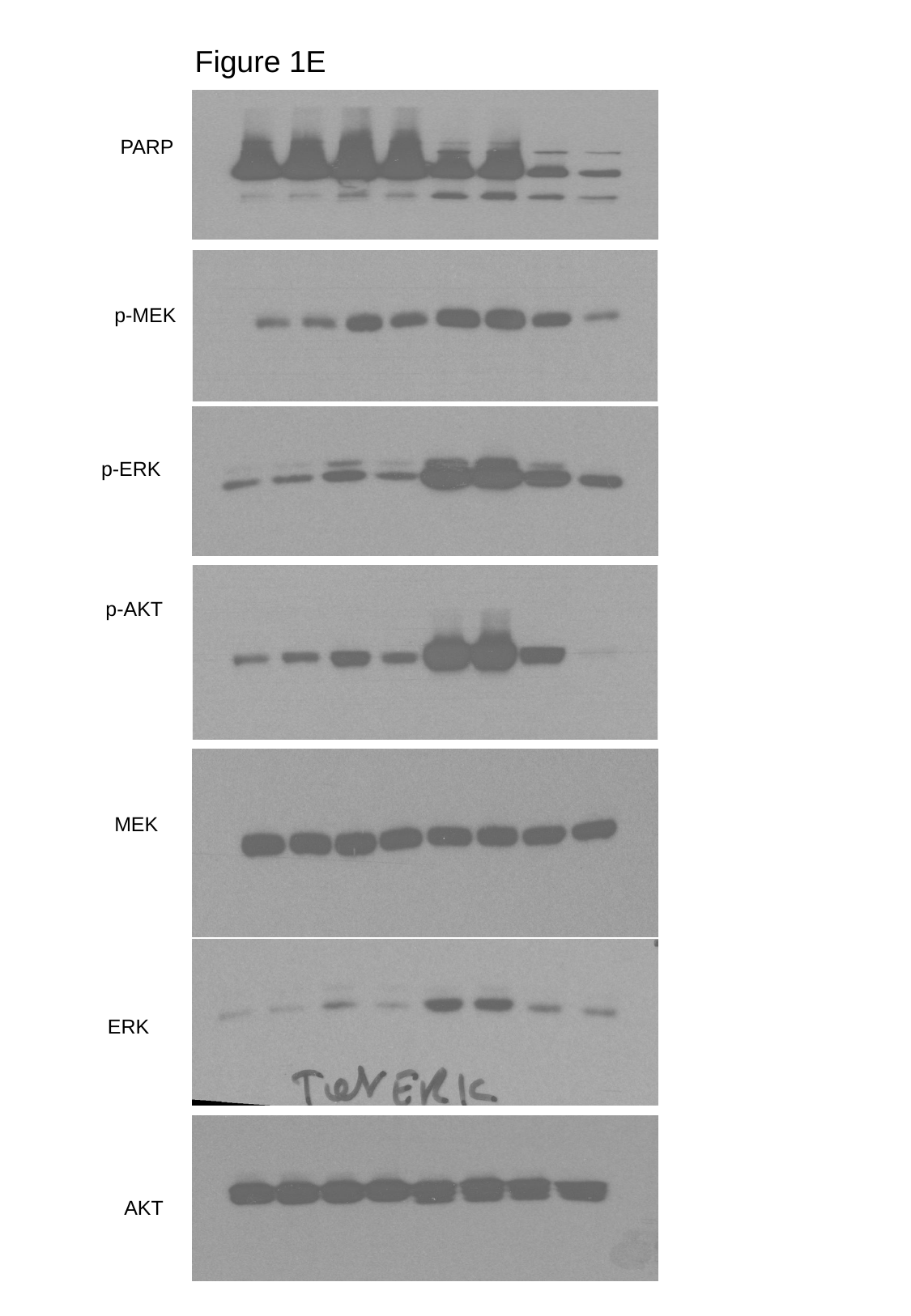

Figure 1E
PARP
p-MEK
p-ERK
p-AKT
MEK
ERK
AKT

## Slide 5
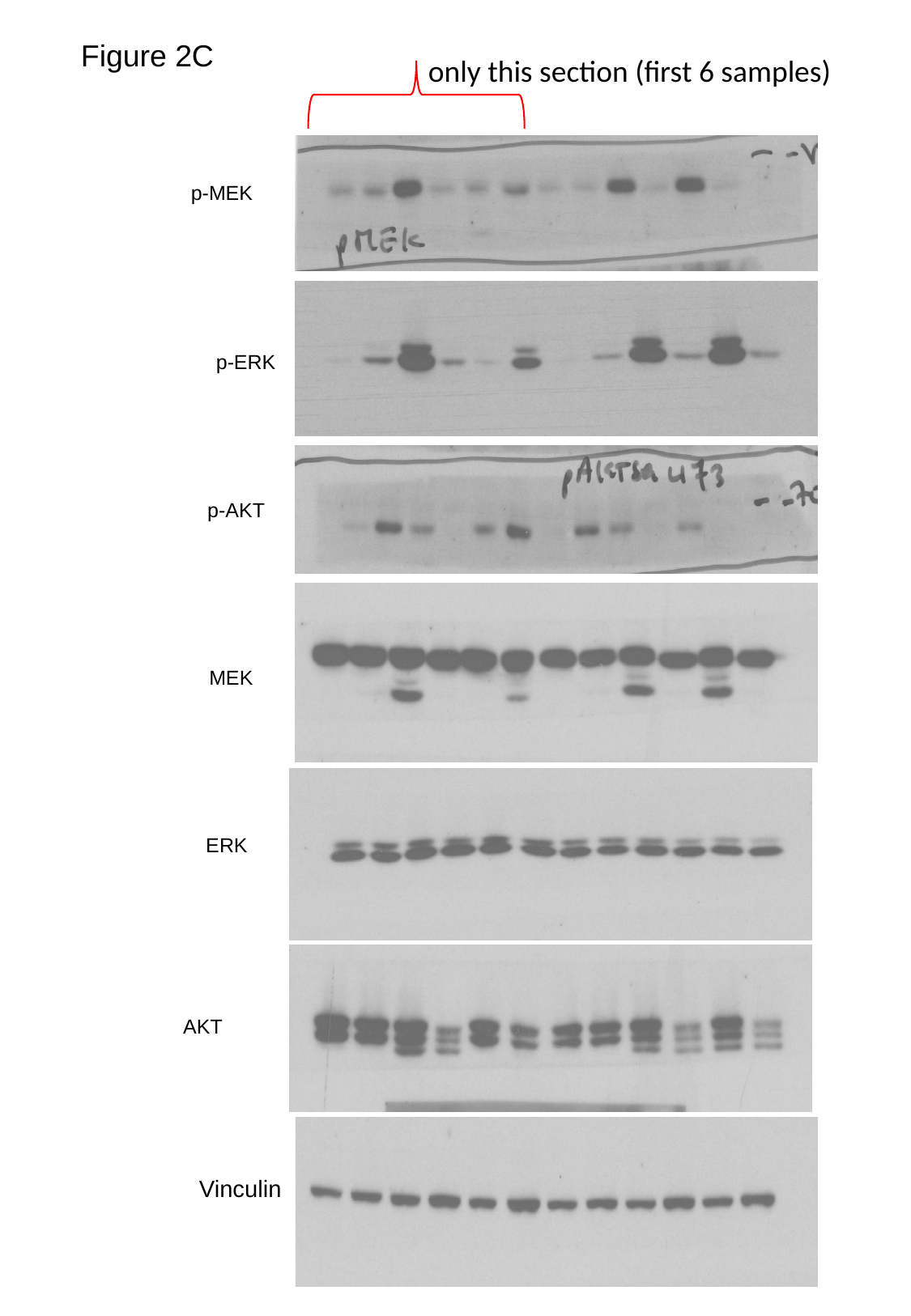

Figure 2C
only this section (first 6 samples)
p-MEK
p-ERK
p-AKT
MEK
ERK
AKT
Vinculin

## Slide 6
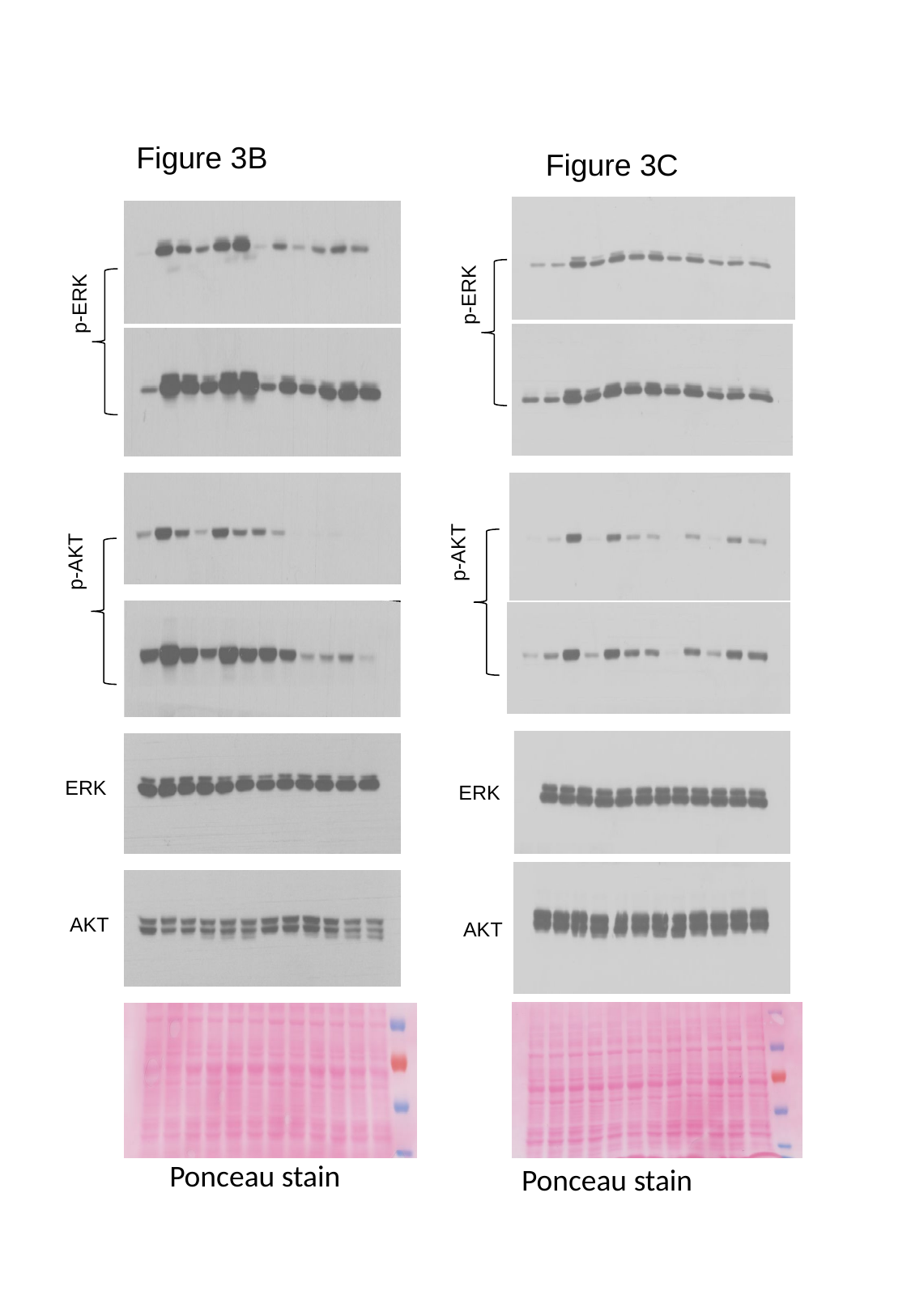

Figure 3B
Figure 3C
p-ERK
p-ERK
p-AKT
p-AKT
ERK
ERK
AKT
AKT
Ponceau stain
Ponceau stain

## Slide 7
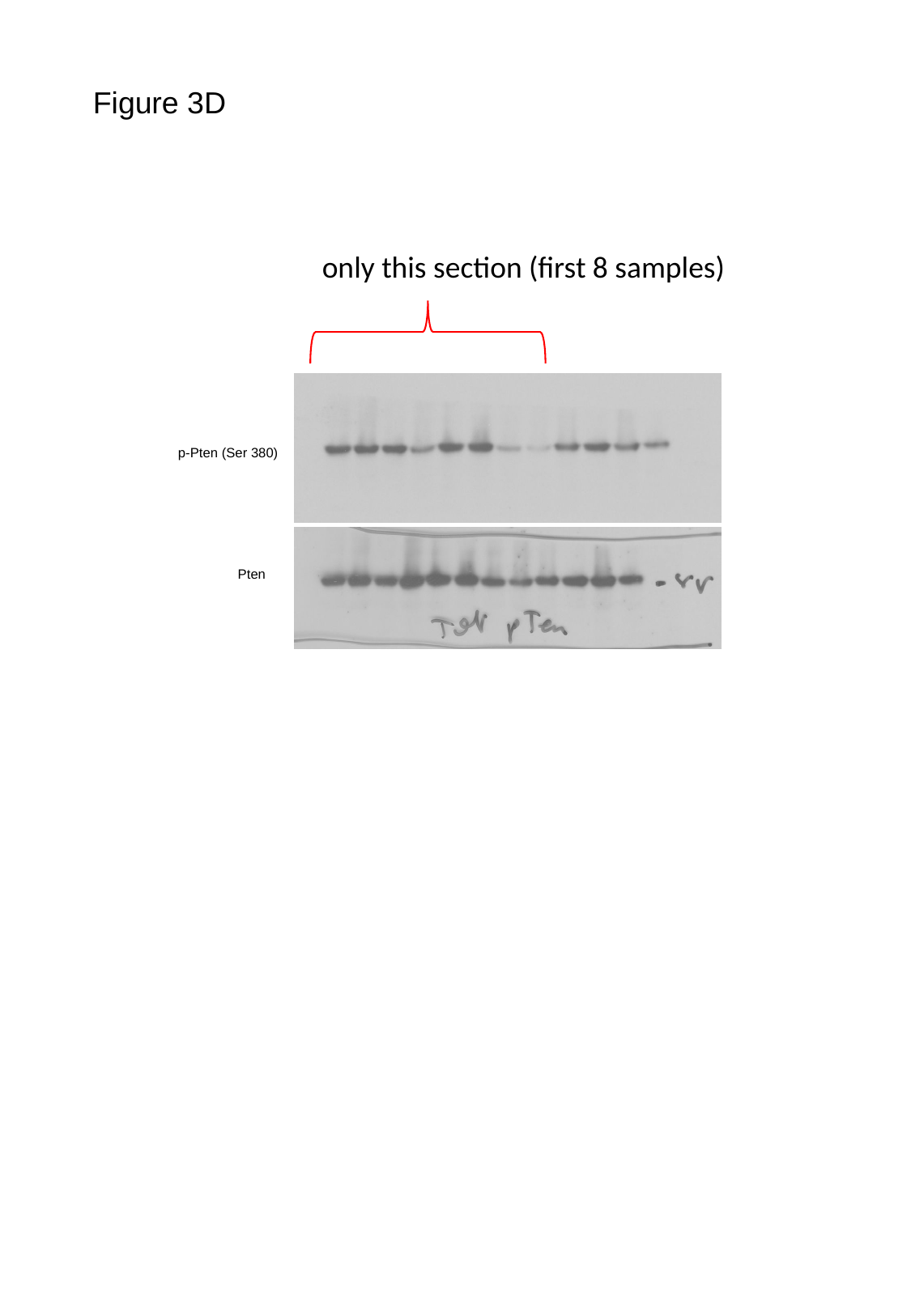

Figure 3D
only this section (first 8 samples)
p-Pten (Ser 380)
Pten

## Slide 8
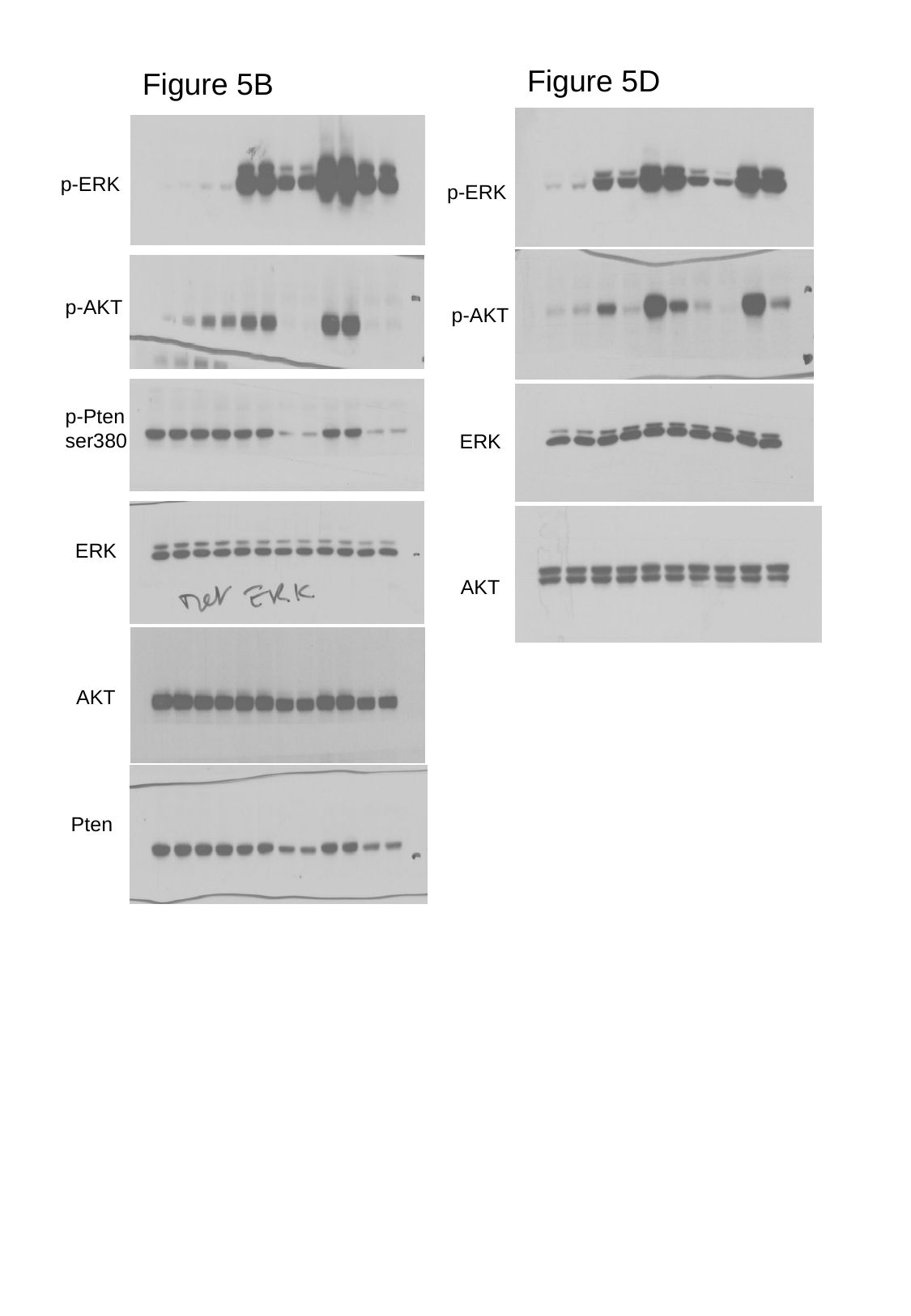

Figure 5D
Figure 5B
p-ERK
p-ERK
p-AKT
p-AKT
p-Pten ser380
ERK
ERK
AKT
AKT
Pten

## Slide 9
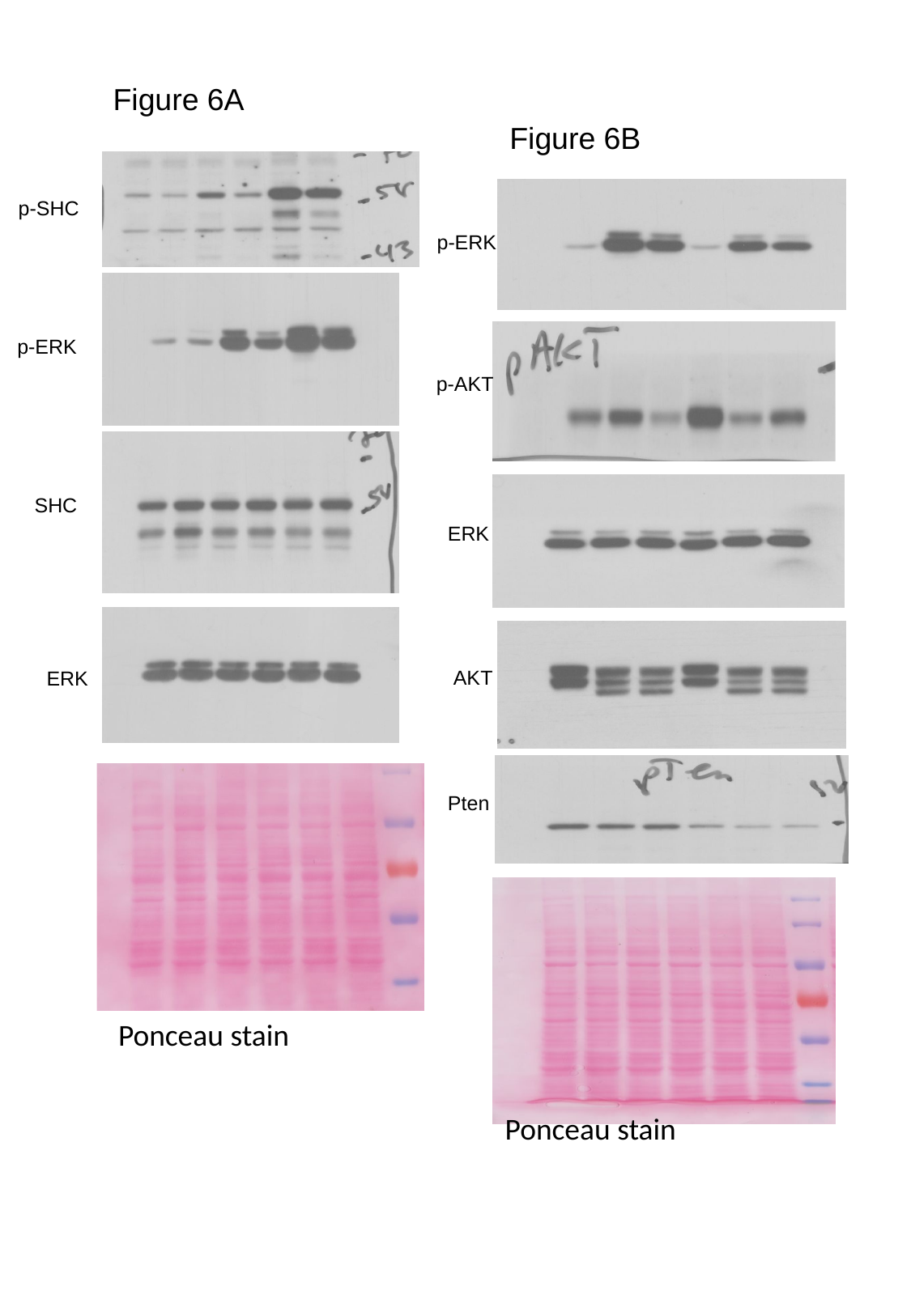

Figure 6A
Figure 6B
p-SHC
p-ERK
p-ERK
p-AKT
SHC
ERK
AKT
ERK
Pten
Ponceau stain
Ponceau stain

## Slide 10
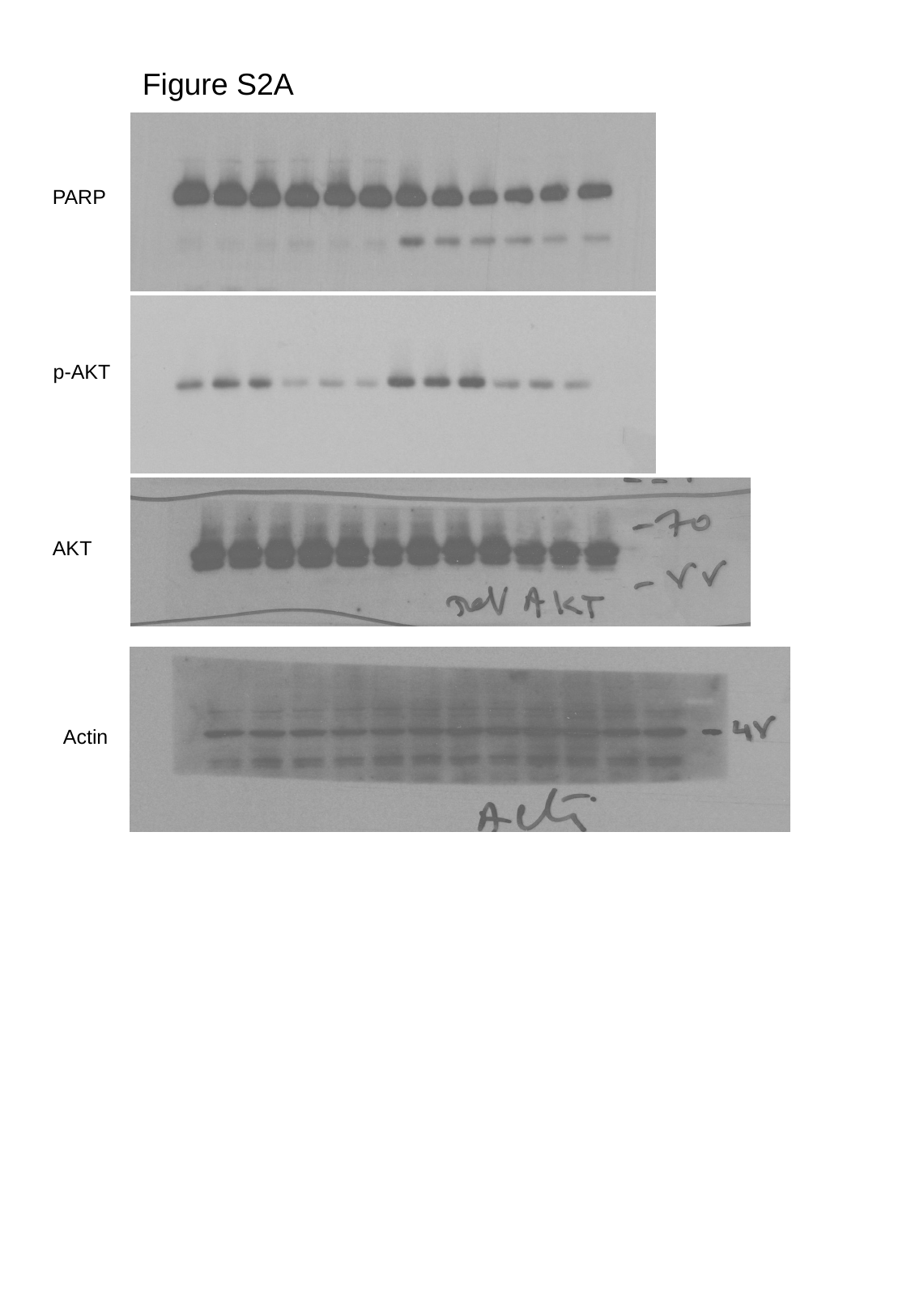

Figure S2A
PARP
p-AKT
AKT
Actin

## Slide 11
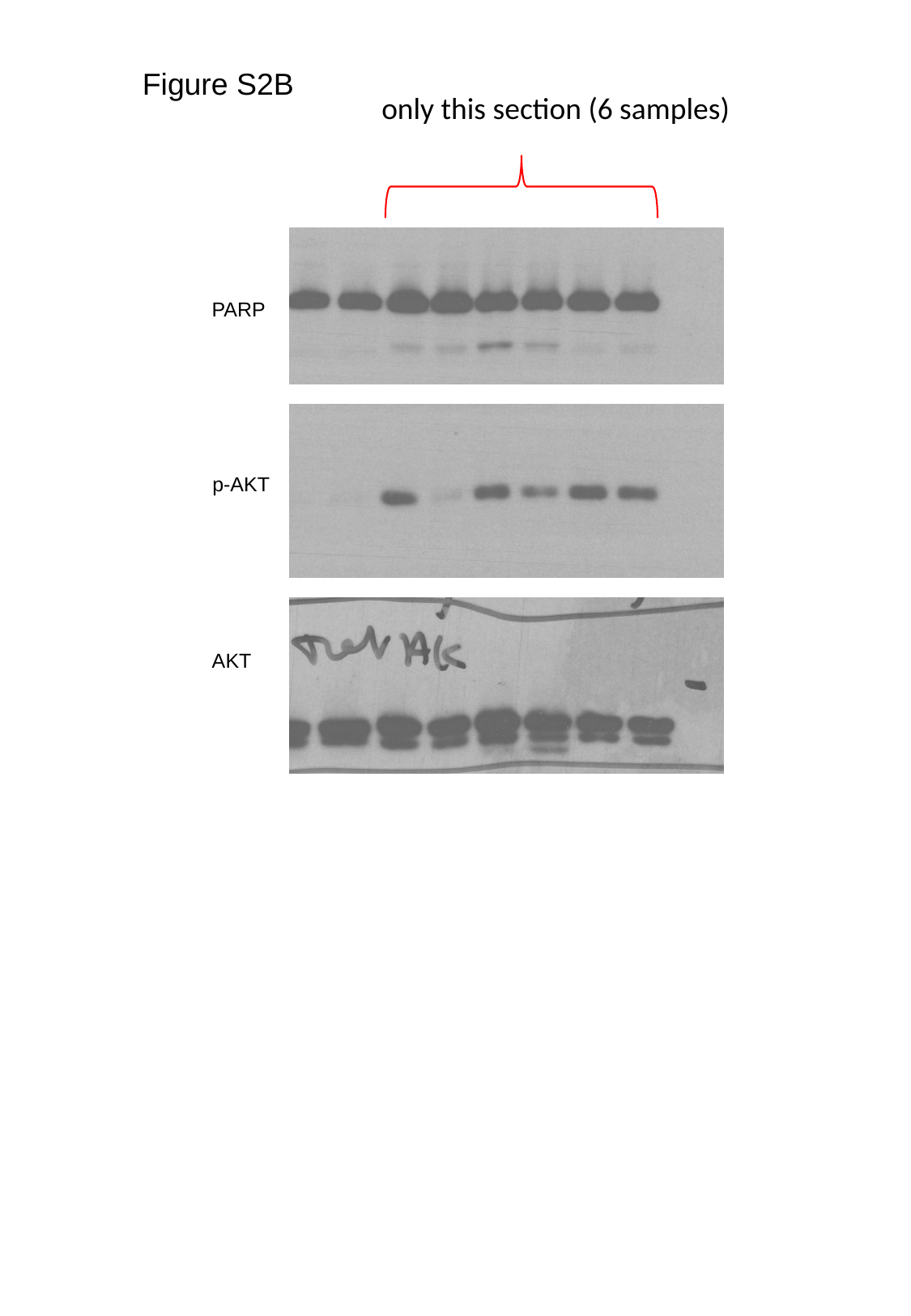

Figure S2B
only this section (6 samples)
PARP
p-AKT
AKT

## Slide 12
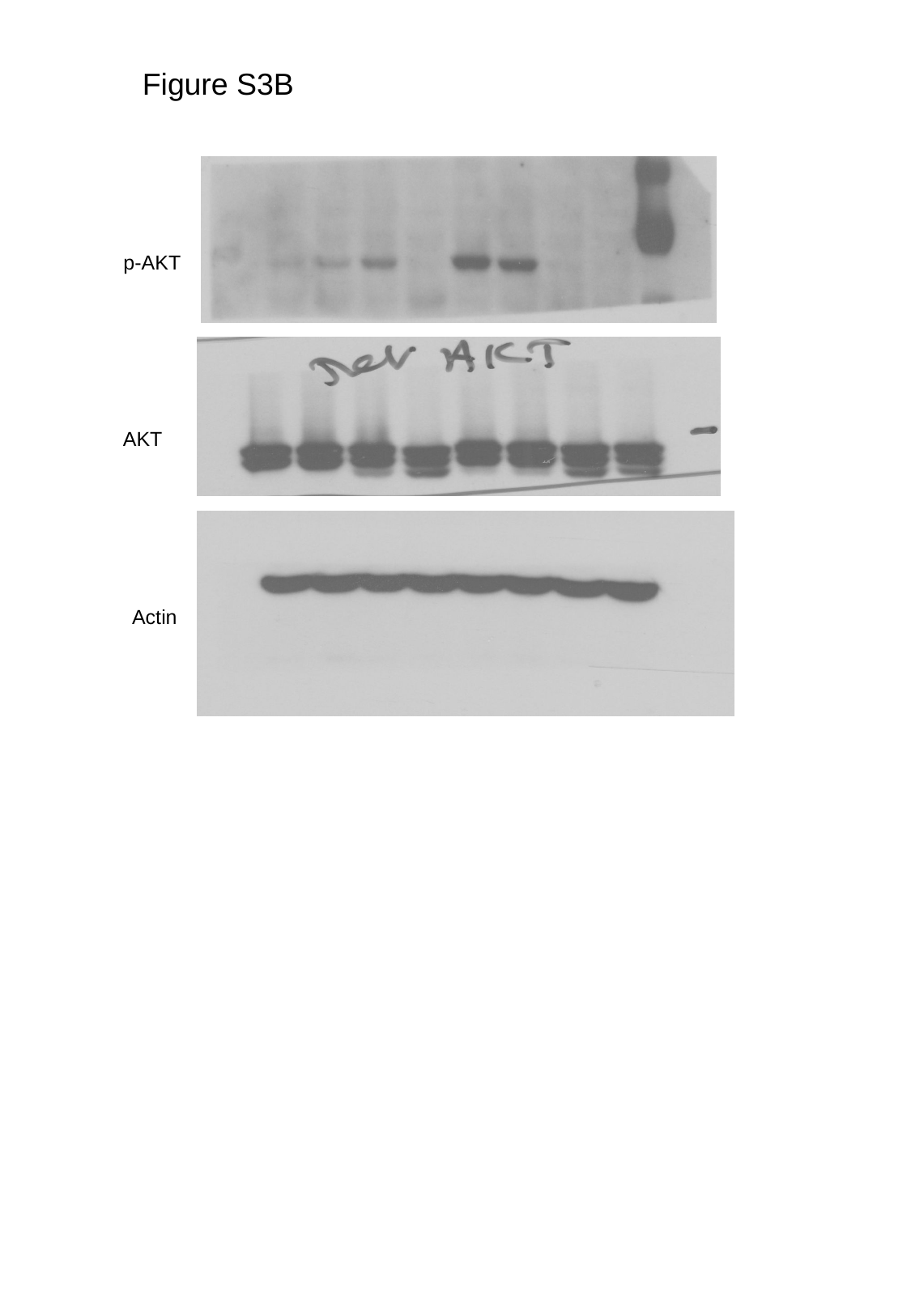

Figure S3B
p-AKT
AKT
Actin
